# Detection of subtle white matter lesions in MRI through texture feature extraction and boundary delineation using an embedded clustering strategy

**DOI:** 10.1038/s41598-022-07843-8

**Published:** 2022-03-15

**Authors:** Kokhaur Ong, David M. Young, Sarina Sulaiman, Siti Mariyam Shamsuddin, Norzaini Rose Mohd Zain, Hilwati Hashim, Kahhay Yuen, Stephan J. Sanders, Weimiao Yu, Seepheng Hang

**Affiliations:** 1 Bioinformatics Institute, A*STAR, Singapore, Singapore; 2 Institute of Molecular and Cell Biology, A*STAR, Singapore, Singapore; 3 Department of Mathematical Sciences, Faculty of Science, Universiti Teknologi Malaysia, UTM Skudai, 81310 Johor, Malaysia; 4 School of Computing, Faculty of Engineering, Universiti Teknologi Malaysia, Johor, Malaysia; 5 Department of Radiology, Hospital Kuala Lumpur, Kuala Lumpur, Malaysia; 6 Department of Psychiatry and Behavioral Sciences, UCSF Weill Institute for Neurosciences, University of California, San Francisco, USA; 7 School of Pharmaceutical Sciences, Universiti Sains Malaysia, Penang, Malaysia; 8 Department of Radiology, Faculty of Medicine, Universiti Teknologi MARA, Sungai Buloh, Malaysia; 9 Computational Digital Pathology Laboratory, Bioinformatics Institute (BII), 30 Biopolis Street, #07-46 Matrix, Singapore, 138671 Singapore

**Keywords:** Computational models, Image processing, Machine learning

## Abstract

White matter lesions (WML) underlie multiple brain disorders, and automatic WML segmentation is crucial to evaluate the natural disease course and effectiveness of clinical interventions, including drug discovery. Although recent research has achieved tremendous progress in WML segmentation, accurate detection of subtle WML present early in the disease course remains particularly challenging. Here we propose an approach to automatic WML segmentation of mild WML loads using an intensity standardisation technique, gray level co-occurrence matrix (GLCM) embedded clustering technique, and random forest (RF) classifier to extract texture features and identify morphology specific to true WML. We precisely define their boundaries through a local outlier factor (LOF) algorithm that identifies edge pixels by local density deviation relative to its neighbors. The automated approach was validated on 32 human subjects, demonstrating strong agreement and correlation (excluding one outlier) with manual delineation by a neuroradiologist through Intra-Class Correlation (ICC = 0.881, 95% CI 0.769, 0.941) and Pearson correlation (*r* = 0.895, *p*-value < 0.001), respectively, and outperforming three leading algorithms (Trimmed Mean Outlier Detection, Lesion Prediction Algorithm, and SALEM-LS) in five of the six established key metrics defined in the MICCAI Grand Challenge. By facilitating more accurate segmentation of subtle WML, this approach may enable earlier diagnosis and intervention.

## Introduction

White matter lesions (WML) are regions of abnormal myelination in the nervous system. WML are often seen as progressive brain changes in the elderly population, where the presence, shape, location and size of WML are important indicators of the etiology of neurological and geriatric disorders, including stroke^1,2^, Alzheimer’s disease^3,4^, vascular dementia^2,3,5^, cognitive decline^6,7^, depression^8–10^, balance, and gait impairment^11,12^. WML may also reflect inflammatory diseases such as multiple sclerosis, tumors, or vascular lesions. Accurate detection of these lesions has the potential to inform the diagnosis of the underlying disorders by characterizing their appearance and distribution, which in turn guides prognostication of the disease course and opportunities for earlier clinical intervention^13,14^. Precise delineation of their boundaries and quantification of disease burden in drug development research^15^ and future clinical settings may also shed light on the underlying disease patterns and evolution, potentially revealing new disease subtypes and serving as pharmacodynamic biomarkers of treatment response.

WML are commonly identified with Magnetic Resonance Imaging (MRI) techniques on Proton Density (PD), T2-weighted (T2-w) spin echo or fast spin echo sequence, and another, complementary T2-based technique, Fluid-Attenuated Inversion Recovery (FLAIR) pulse sequences. In particular, FLAIR MRI shows WML prominently as diffuse and bright (hyperintense) regions in the brain, and thus WML are also known as white matter hyperintensities. Subsequently, diffusion and magnetization transfer^16,17^ MRI serve as secondary sequences to identify active (acute) disease within WML. The assessment of white matter lesions is a tedious and challenging process for neuroradiologists. Common visual rating scales such as the Scheltens scale^18^, the Age-Related White Matter Changes (ARWMC) scale^19^, and the Fazekas scale^20^ are manual and subjective, dependent on the neuroradiologist’s experience, interpretation, and judgment, and high intra- and inter-subject variability in WML assessment among neuroradiologists has impaired reproducibility^21^. To address these limitations, quantification of WML volume using computer-aided detection and diagnosis techniques can assist neuroradiologists with objective, automated assessments that more reliably capture the extent and progression of WML in patients. On FLAIR MRI, WMLs seen in multiple sclerosis (MS) exhibit similar characteristics to WMLs seen in ischaemia, allowing similar analytic methods to be used for both.

Developing a computer-aided detection method to delineate WML is a challenging task, as the size, shape, and location of these lesions vary from subject to subject, further confounded by MRI flow artefact and image noise. Many WML segmentation methods have been developed to address these challenges^22^, including Trimmed Mean Outlier Detection (TMOD) by our group^23^. TMOD was evaluated on the MS data set from the MICCAI Challenge^24^, where it ranked third compared to other methods with a total score of 81.95 at the time of submission^23^. Roura et al.^25^ introduced the SALEM-LS (SLS) algorithm, which uses an adaptive outlier algorithm to threshold outliers as WML from grey matter, ranking first on this dataset with a total score of 82.34. The Lesion Prediction Algorithm (LPA) developed by Schmidt et al.^26–28^ was based on logistic regression and successfully applied to the longitudinal analysis of WML recently^29^. Vanderbecq and colleagues^30^ recommended both the SLS and LPA algorithms as reasonable first choice WML segmentation tools after comparison and validation of seven different algorithms in their recent study.

Taking a similar approach to TMOD, Wu et al.^31^ used intensity histograms of FLAIR images for WML detection and adapted fuzzy connectedness for segmentation^32,33^. De Boer et al.^34^ also used histogram thresholds to detect WML. Thresholds were adjusted adaptively based on grey matter voxel distribution, defined by T = μ + ασ, where the α was an optimized threshold parameter that was computed based on the highest similarity index from a set of ground truth annotated by a neuroradiologist. A recent study by Guizard et al.^35^ used a supervised method using non-local means to segment MS lesions leading to accurate identification and segmentation of MS lesions regardless of lesion orientation, size, and shape. In the same year, a Bayesian model applied to WML segmentation was introduced by Sudre et al.^36^. The main strength of this method was its ability to distinguish different types of abnormal brain tissue without prior pathological knowledge. Distinguishing different types of outliers, such as iron deposition, from WML helps improve diagnosis and management of age-related cognitive decline. These fully automated approaches have the advantage of reducing inter- and intra-rater variability through an objective, consistent analysis after training on a large body of images, at least in theory. With these advancements, fully automated methods have become a preferred scheme for neuroradiologists to analyze and quantify WML in large MRI datasets, especially for longitudinal progression of WML in developmental studies, where large numbers of subjects with subtle changes across time require sensitive, high-throughput techniques.

One should note that false positive (FP) detection is a common problem detracting from WML segmentation accuracy^30,37–39^. FP can arise from inadvertent segmentation of image noise, confounding non-brain structures such as signal from the skull and the optic nerve after inadequate skull-stripping, and other image artifacts^40^ that can appear hyperintense in the FLAIR modality. Although such hyperintensities are often clearly identifiable as non-WML to the human eye, automated algorithms may have difficulty distinguishing these hyperintensities from WML, leading to false positive signals. This distinction becomes especially challenging for the subtle (spotted or punctate) WML seen in the mild WML loads of early disease, typically < 5 mL. Several studies^39,41^ have shown that these subtle WML are extremely common during aging, merging together over time to become confluent WML. In a very recent study of neuropathological changes^6^, punctate WML associated with white matter microvascular networks likely reflect underlying white matter disease such as myelin damage, glial hyperplasia, and increased perivascular space. These punctate lesions seen in FLAIR images may be among the earliest indicators of this disease, and early intervention may reduce disease progression^14,42^. Simple morphological operation and rule-based methods are applied in post processing steps to reduce FP, but the results are insufficient^43^, especially for ill-defined, subtle lesions.

**Figure 1:**
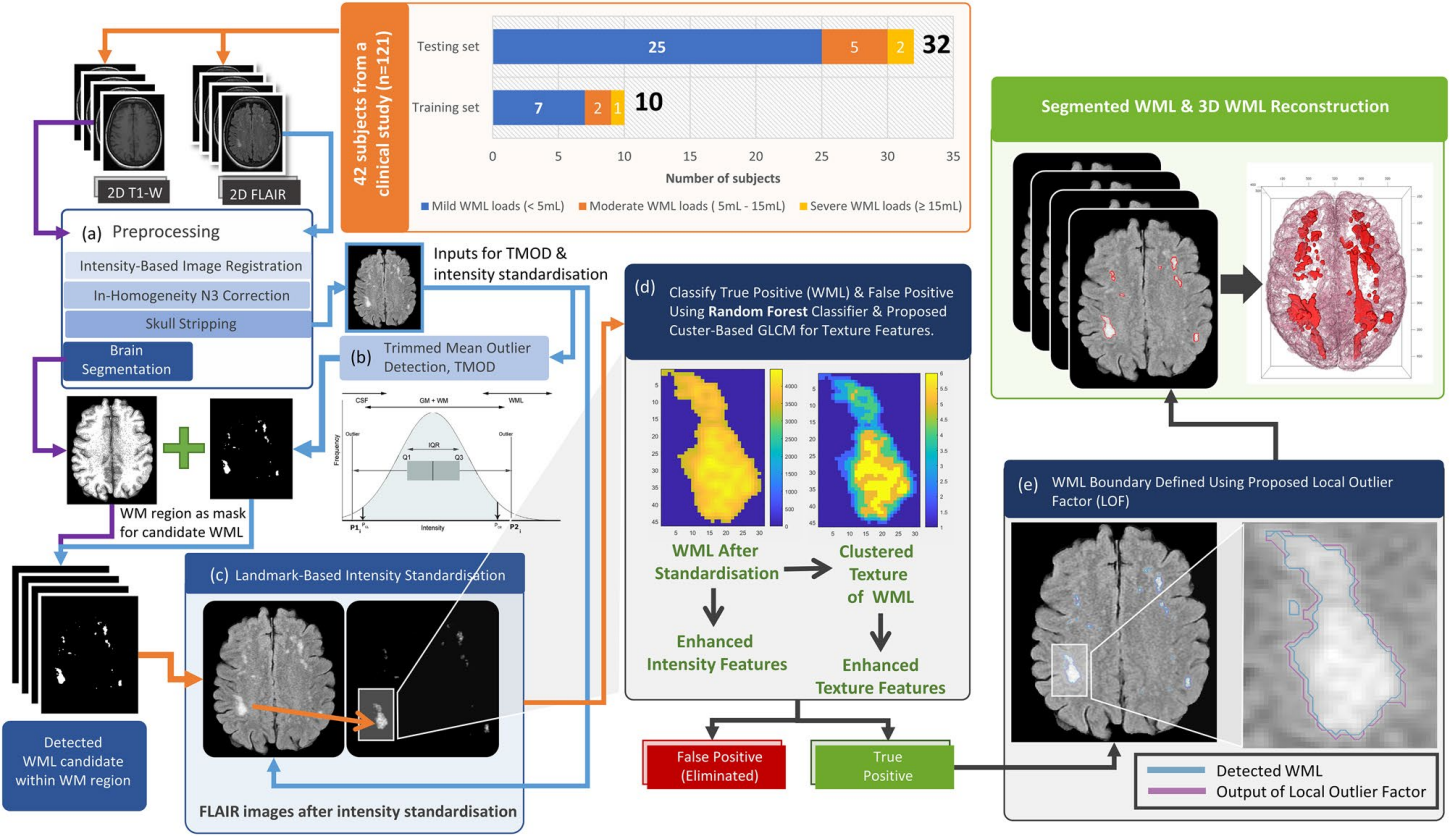
Flow chart of the proposed method. (**a**) The method used for image preprocessing. (**b**) The method used to detect WML candidates. (**c**) The method used for FLAIR image intensity standardization. (**d**) The method used to detect WML (true positive) from WML candidates. (**e**) Refining the boundaries of WML segmentation.

Here, we build upon the TMOD approach to minimize the number of FP WML and refine WML boundary delineation to improve segmentation of mild WML loads (< 5 mL) for early detection of abnormalities that may herald underlying disease. We extract texture features critical to defining the morphology of true white matter lesion using intensity standardization coupled with gray level co-occurrence embedded clustering, followed by Random Forest (RF) based classification of these features to substantially reduce these false positives. We further refine the boundaries of these WML using a Local Outlier Factor (LOF) algorithm^44^ to identify edge pixels more precisely, thus detecting both lesions and their shapes more accurately. The overview of the proposed framework is illustrated in Fig. 1. We validate the method on 32 scans and compare our approach with three other leading algorithms, the Trimmed Mean Outlier Detection^23^, Lesion Prediction Algorithm (LPA)^27^, and SALEM-LS (SLS)^25,45^ methods, to evaluate their performance, which demonstrates that our approach correlates the closest with manual WML delineation by a neuroradiologist.

## Results

To improve the TMOD algorithm, we first established a ground truth dataset using manual WML segmentation by an experienced neuroradiologist on 2-dimensional axial FLAIR MRI images from 32 subjects across a range of WML loads, independently verified by a second neuroradiologist. Representative images with three different levels of two-dimensional and three-dimensional WML segmentation images can be seen in Sect. 1 of the Supplementary Material.

**Figure 2:**
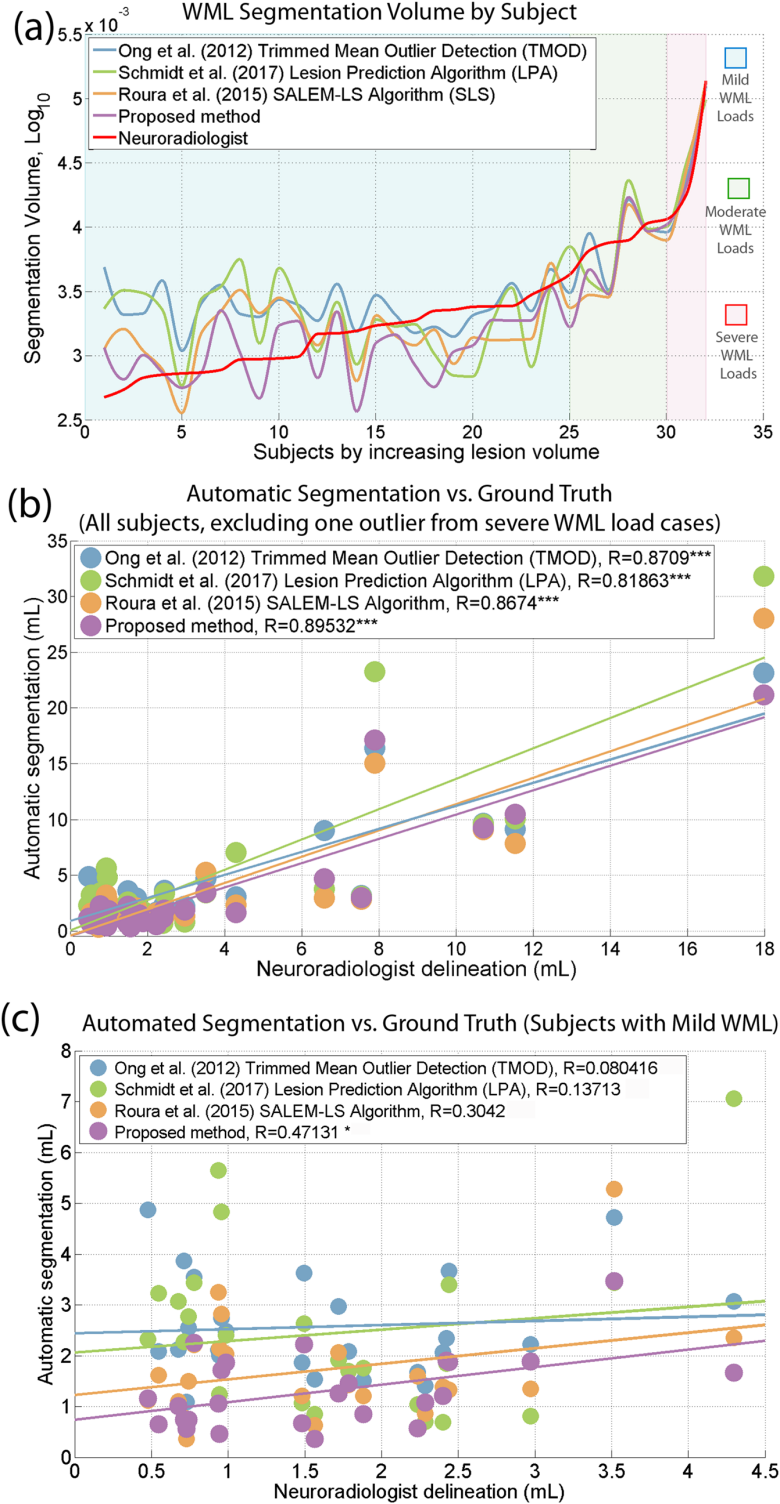
Correlations between automated WML segmentations and ground truth stratified by lesion load. (**a**) The total WML load for each subject manually segmented by the neuroradiologist and automated methods was quantified and sorted by increasing lesion volume in the corresponding ground truth. Subjects were stratified into three WML load groups: mild (< 5 mL, blue background), moderate (5–15 mL, green background) and severe (> 15 mL, red background), also based on the ground truth lesion load. (**b**) A scatter plot of the simple linear regression analysis (32 subjects minus one outlier from severe WML load cases) for WML volume of each automated method compared to the ground truth. (**c**) A scatter plot of the simple linear regression analysis (25 subjects with < 5 mL WML load) for each automated method compared to the ground truth.

We quantified and compared the total WML load of each subject through manual segmentation, our revised method, and the three state-of-the-art WML automatic segmentation methods: TMOD, SLS, and LPA (see Fig. 2a, sorted by increasing lesion volume of ground truth)^23,26,45^. We chose TMOD as our prior method, verified on the MICCAI Challenge MS dataset, and SLS as the first ranking algorithm using the same dataset. We also selected LPA as the recommended first choice WML segmentation algorithm along with SLS by Vanderbecq et al.^30^. We applied the open-source implementations of each algorithm (available for free in the scientific community), and Sect. 2 of the supplementary material provides additional explanations about the parameter settings used in each method.

Spearman correlation coefficient and Intra-Class Correlation coefficient (ICC) were employed to measure the correlation and agreement (excluding one outlier), respectively, between the WML load (volume, mL) detected by each algorithm with manual delineation by an experienced neuroradiologist. The Pearson correlation coefficient showed a significant, strong correlation (*r* = 0.895, *p*-value < 0.001) between the proposed method and manual delineation by a neuroradiologist as illustrated in Fig. 2b. Although all algorithms showed high and relatively similar *r* values, demonstrating overall strong correlation with WML across all sizes, that of the proposed method was numerically the highest (vs. TMOD: *r* = 0.871, LPA: *r* = 0.819, SLS: *r* = 0.867).

As an additional reliability test, we obtained the ICC coefficient to evaluate the agreement between the manual segmentation by radiologists and automated segmentations. An appropriate ICC for this reliability analysis was chosen based on the procedure recommended by Ko and Li^46^, the single measurement reliability with “absolute agreement” since there are two raters (manual and automated segmentation). The ICC with the proposed method is 0.882 (95% CI: 0.769, 0.941), the strongest agreement among the evaluated methods (vs. TMOD: ICC = 0.840, 95%, CI 0.671, 0.923; LPA: ICC = 0.711, 95%, CI 0.483, 0.849; SLS: ICC = 0.832, 95%, CI 0.680, 0.915).

The low WML load and scattered distribution of the WMLs early in disease processes is particularly challenging for automated WML detection and segmentation, with many algorithms leading to multiple FPs. To interrogate these lesions, we stratified the lesion load into three previously defined groups^28,47,48^: mild (< 5 mL), moderate (5–15 mL), and severe (> 15 mL) based on the ground truth lesion load. In addition to evaluation of gross WML loads, we measured the quality of segmentations to evaluate their morphological accuracy. The evaluation of WML segmentation quality is varied in the literature. Evaluation metrics (see Table 1) were chosen based on the well-known metrics defined in the MICCAI Grand Challenge on MS Lesions Segmentations^24^ and summaries by Geremia et al.^49^ with a similar aim of segmenting WML accurately based on symmetrical features. Together these sources yielded six evaluation metrics to evaluate segmentation performance: Dice index (DI), Jaccard index (JI), positive predictive value (PPV), volume difference (VD), false positive rate (FPR), and true positive rate (TPR) to compare the agreement between automated segmentations and ground truth.

**Table 1:** The six evaluation metrics are chosen from the conventional and well know evaluation parameters presented in the MICCAI grand challenge and literature. The Dice index (DI), Jaccard index (JI), and volume difference (VD) are used to measure the quality of WML segmentation by calculating their volume and Euclidean distance of WML boundary. Whereas, the true positive rate (TPR), false positive rate (FPR) and positive predictive value (PPV) usually practice understanding the quality of WML detection.

Evaluation metrics	Definition	Best	Worst
DI	$\frac{2\times Vol({S}_{auto})\cap Vol(GT)}{Vol({S}_{auto})+Vol(GT)}$	1	0
JI	$\frac{2\times Vol({S}_{auto})\cap Vol(GT)}{Vol({S}_{auto})\cup Vol(GT)}$	1	0
TPR	$\frac{TP}{TP+FN}$	1	0
FPR	$\frac{FP}{TP+TN}$	0	1
PPV	$\frac{TP}{TP+FP}$	1	0
VD	$\frac{Vol({S}_{auto})-Vol(GT)}{Vol(GT)}$	0	∞

The notations for Vol (${S}_{auto}$) is volume of WML segmented by automated method, Vol ($GT$) is volume of WML delineated by neuroradiologist, TP is number of true positive, FP is number of false positive, FN is number of false negative, and TN is number of true negative. ﻿The best is defined as perfect segmentation and worst is completely missed segmentation.

**Figure 3:**
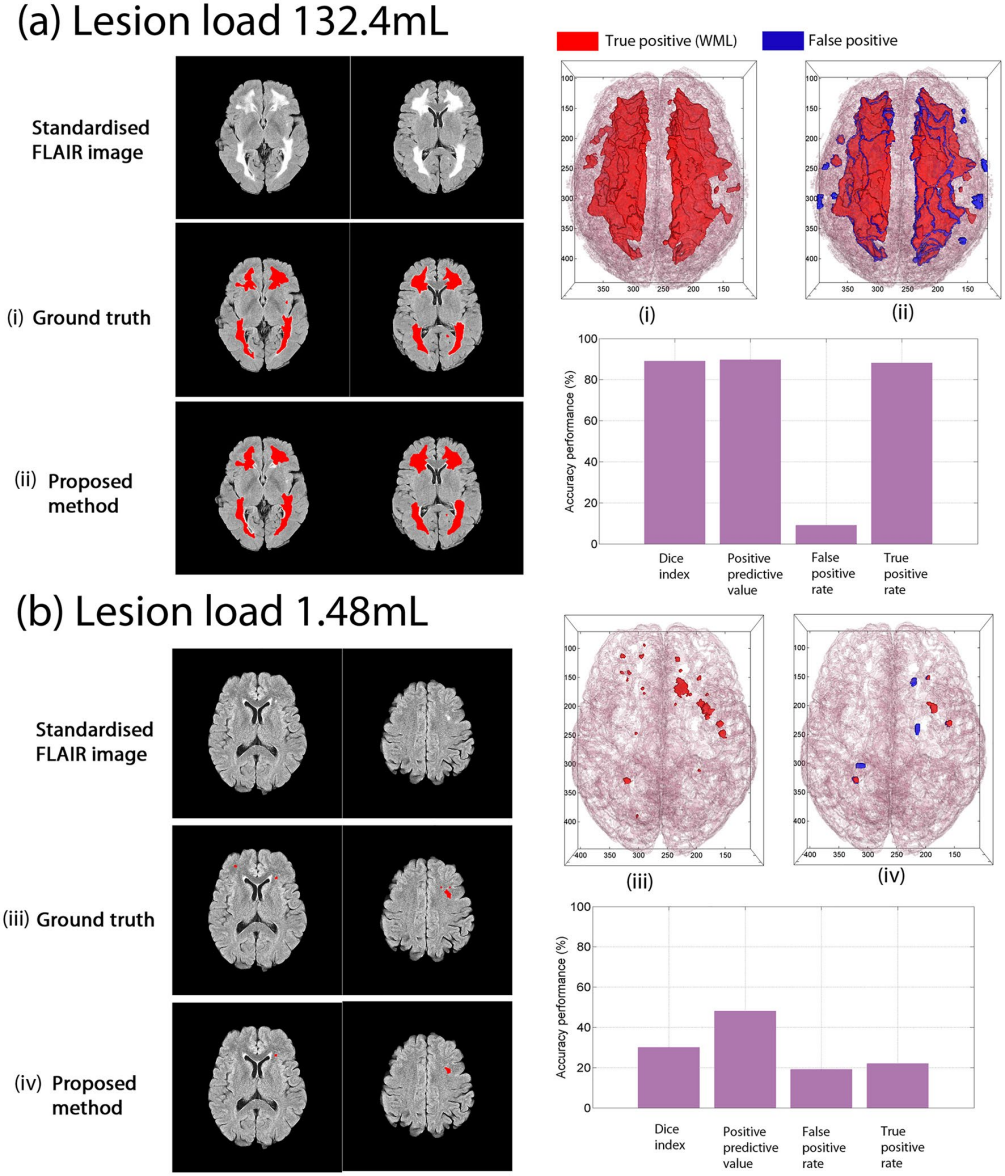
Comparison of the proposed method to ground truth for severe and mild cases. Representative examples of the performance of the proposed WML detection and segmentation are shown, with several slices of a WML binary image results segmented by a neuroradiologist and segmented by the proposed method superimposed on top of the intensity standardised FLAIR image. The binary image data of white matter and white matter lesions were also used for three-dimensional reconstruction. (**a**) For severe cases, the results of both are very close. (**b**) The results of mild cases show greater discrepancy, which is also the challenge of the proposed method and other comparative methods for mild cases.

Qualitatively, we observed accurate performance of the proposed method in detecting and segmenting WML in images across WML loads (Fig. 3), reinforced by quantitative assessments. For a representative subject with high WML load (132.4 mL, Fig. 3a), the Dice Index is 89%, PPV is 90%, TPR is 88% and FPR is 9%, in line with previous studies for high WML loads (> 15 mL), where the DI, PPV, and TPR values are typically above 80% (TPR for TMOD: 84%, LPA: 70%, SLS: 91%). By contrast, the FPR value obtained by the proposed method is less than 10%, also in line with the other methods (TMOD: 6%, LPA: 0.9%, SLS: 7%). These high accuracy results are as expected because the lesions are prominent, and the area is large.

**Figure 4:**
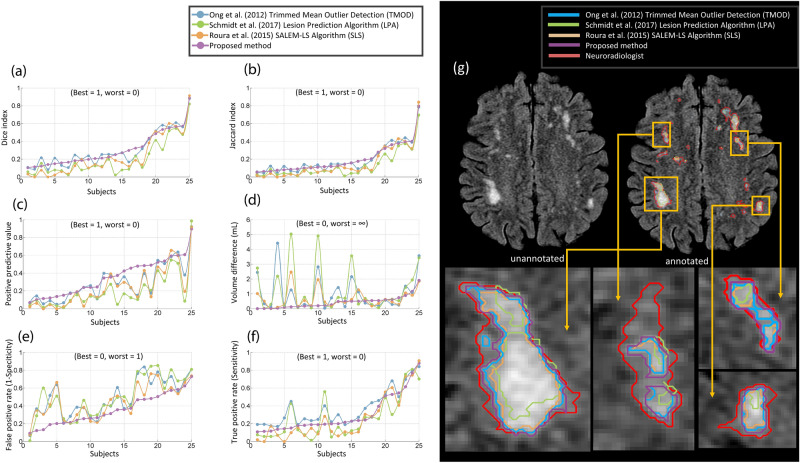
The performance of the proposed method compared to TMOD, LPA and SLS in well-established evaluation metrics. They are (**a**) Dice index, (**b**) Jaccard index, (**c**) positive predictive value, (**d**) volume difference, (**e**) false positive rate and (**f**) true positive rate. (**g**) The comparison of the proposed method and other comparative methods by qualitative analysis of WML segmentation.

Given the importance and challenge of identifying and segmenting mild WML loads in subjects for earlier diagnosis, we further focused on the accuracy of segmentation performance with WML loads < 5 mL. For these loads, the proposed method is the only one that showed a positive, statistically significant correlation (*r* = 0.471, *p*-value < 0.05, *n* = 25 subjects, see Fig. 2c, vs. TMOD: *r* = 0.080, LPA: *r* = 0.137, SLS: *r* = 0.304). Compared with the higher loads, the accuracy metrics are much lower for mild loads, where the Dice Index is 30%, PPV is 48%, TPR is 22%, and FPR is 19% (see Fig. 3b), in line with previous studies^25,50,51^. Figure 4a,b illustrate the Dice Index and Jaccard Index, respectively, as line plot analyses, and Table 2 shows the summarised means and standard deviation of four of the essential agreement measures of WML segmentation for the mild WML group, which show generally improved and stabler metrics compared with the other methods. Notably, the proposed method received the highest Dice Index (26.5%) for the average of 25 subjects compared to the TMOD (24.0%), SLS (14.7%, *p*-value ≤ 7.19 × 10^–4^, Bonferroni corrected for four comparisons), and LPA (12.8%, *p*-value ≤ 1.51 × 10^–5^, Bonferroni corrected). Also, the proposed method showed a substantial, statistically significant reduction in FP with an average FP of 36.6% compared to 55.9% for TMOD (*p*-value ≤ 1.85 × 10^–4^, Bonferroni corrected), 47.5% for SLS (*p*-value ≤ 1.27 × 10^–2^, Bonferroni corrected) and 54.2% for LPA (*p*-value ≤ 1.20 × 10^–3^, Bonferroni corrected). Together with the volume difference metric, these similarity metrics indicate that the proposed method is robust in segmenting mild load volumes along with TMOD and LPA while obtaining a statistically significant improvement in accuracy.

**Table 2:** Agreement measures for Dice index, Jacarrd index, positive predictive value, true positive rate, false positive rate, and volume difference between lesions segmented by the automated methods and a neuroradiologist for 25 subjects with mild WML loads.

Automated segmentation methods	Dice index (B = 1, W = 0)	Jaccard index (B = 1, W = 0)	Positive predictive value (B = 1, W = 0)	True positive rate (B = 1, W = 0)	False positive rate (B = 0, W = 1)	Volume difference (B = 0, W = ∞)
Proposed method	0.2648 ± 0.134	0.1598 ± 0.10	0.3383 ± 0.218	0.2555 ± 0.143	0.3656 ± 0.189	0.5056 ± 0.435
TMOD	0.2397 ± 0.123 NS	0.1420 ± 0.09 NS	0.2180 ± 0.139 **	0.3351 ± 0.150 *	0.5594 ± 0.191 ***	1.4350 ± 2.005 **
SLS	0.1471 ± 0.131 ***	0.0851 ± 0.09 **	0.1680 ± 0.167 ***	0.1609 ± 0.148 **	0.4748 ± 0.196 *	0.7978 ± 0.653 *
LPA	0.1275 ± 0.080 ***	0.0700 ± 0.05 ***	0.1331 ± 0.122 ***	0.1913 ± 0.157 *	0.5417 ± 0.229 **	1.4973 ± 1.673 **

Dice index, Jaccard index, positive predictive value, true positive rate, false positive rate, and volume difference are presented as mean ± standard deviation. B indicates Best, and W is Worst. NS is not statistically significantly different compared with the proposed method (P > 0.05); * is significant at P ≤ 0.05; ** is significant at P ≤ 0.01, and *** is significant at P ≤ 0.001. All paired t-tests were Bonferroni corrected for four comparisons.

On a per-voxel basis, the proposed method detected WML with a low PPV, or precision, of 33.8% on average, but statistically significant improvement compared to 21.8% for TMOD (*p*-value ≤ 6.10 × 10^–3^, Bonferroni corrected for four comparisons), 16.8% for SLS (*p*-value ≤ 8.07 × 10^–4^, Bonferroni corrected) and 13.3% for LPA (*p*-value ≤ 3.82 × 10^–5^, Bonferroni corrected; see Table 2; Fig. 4c). The true positive rate, or sensitivity, was similarly low for all methods (see Fig. 4f). While per-voxel metrics showed room for improvement in all methods, the proposed method showed close correspondence with ground truth in overall volume differences, having among the lowest differences among all methods across all WML loads, especially mild loads. The mean volume difference was 0.506 mL, substantially reduced compared to 1.435 mL for TMOD (*p*-value ≤ 7.0 × 10^–3^, Bonferroni corrected for four comparisons), 0.798 for SLS (*p*-value ≤ 1.71 × 10^–2^, Bonferroni corrected) and 1.497 mL for LPA (*p*-value ≤ 1.50 × 10^–3^, Bonferroni corrected; see Table 2; Fig. 4d). Additionally, the proposed method showed the best PPV and FPR among all automated methods across the vast majority of WML loads (see Fig. 4c,e).

## Discussion

White matter lesion (WML) segmentation has the potential to play an important role in detection and diagnosis of many neurological disorders. Extending automated WML detection methods to reliably detect lesions in individuals with early-stage disease and milder WML burden (< 5 mL) could improve early diagnosis and act as a pharmacodynamic biomarker. However, to date, such methods have suffered from high false positive rates. Here, we refine the TMOD method to enhance the delineation of mild WML in imaging data from 32 subjects with subtle WML loads. Specifically, the proposed method improves the lesion detection rate by using intensity standardization to capture the full set of intensity feature data, one critical component of the classification model. Similarly, applying the cluster-based GLCM method allows better discrimination of texture features matching those in WML (see Figs. 1d, 6). We demonstrate improved WML segmentation across all state-of-the-art methods as shown qualitatively (see Fig. 4g) and quantitatively, including 2.5 times improved volume difference compared with our prior work and the best ranked performance among the other state-of-the-art methods in the false positive rate, Dice index, positive predictive value, and volume difference for mild WML (see Fig. 4a–f and Table 2). By enhancing subtle WML detection sensitivity while simultaneously and rigorously filtering out FP, the proposed method provides the capability for more effective and accurate early detection and quantification of WML in age-related human disease.

**Figure 5:**
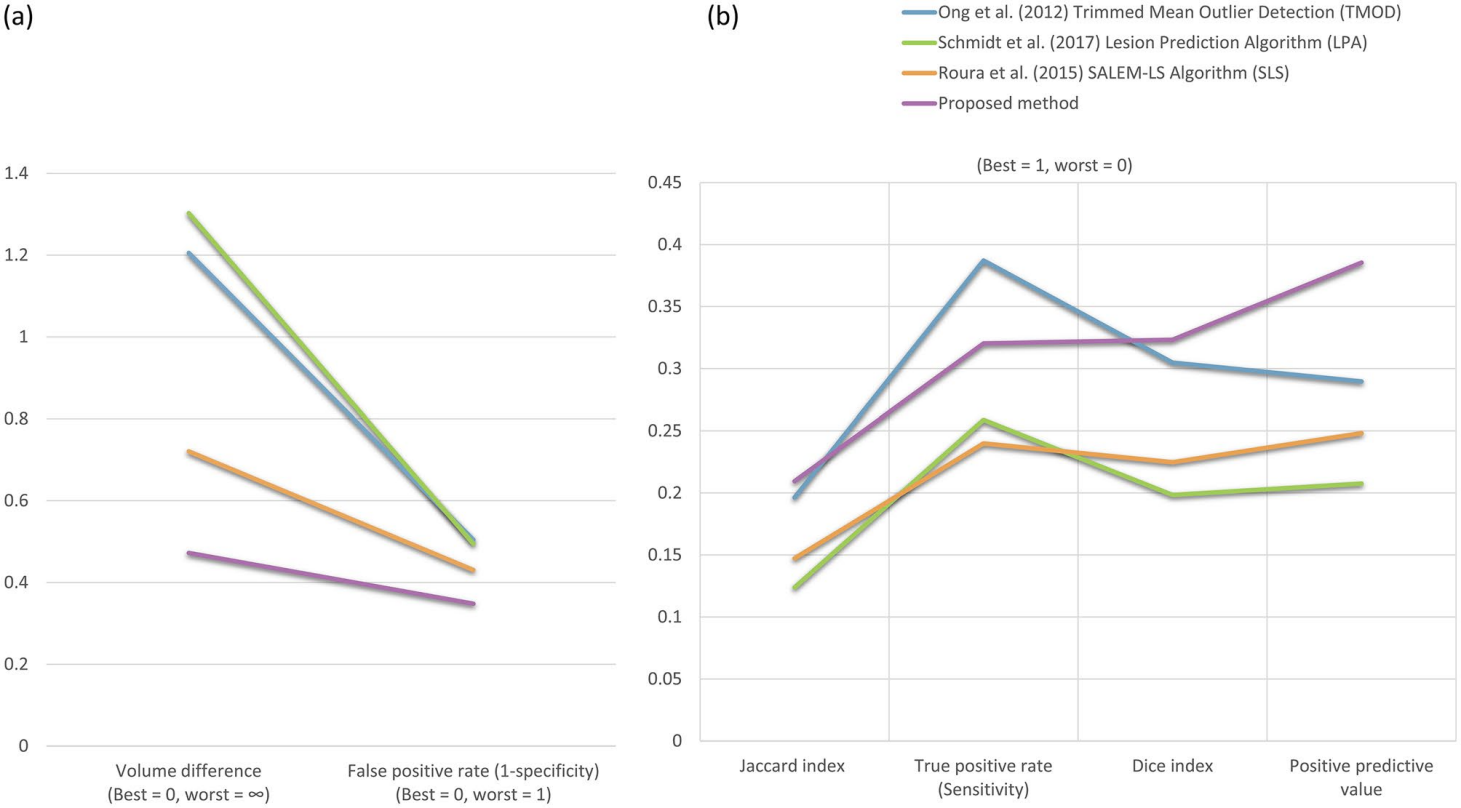
Summaries of all metric evaluations from each automatic method (all 32 subjects). (**a**) Metrics ranging from 0 as the best to 1 or ∞ as the worst performance. (**b**) Metrics with the opposite orientation, where the best performance index is scored 1.0, and the worst performance is 0.

To provide a consistent performance comparison across all state-of-the-art methods for 32 subjects, we found that the proposed method showed the optimal performance for each metric except for sensitivity (see Fig. 5), where it took second to our prior work, trading some sensitivity for increased precision (PPV). Together, the proposed method outperformed the three other automatic methods in measures related to segmentation precision and specificity (the positive predictive value and false positive rate, respectively), critical to identifying relevant lesions for clinical follow-up and monitoring. Furthermore, the proposed method identified lesion patterns with greater fidelity to ground truth (Dice index, Jaccard index, and volume difference), consistent with improved WML boundary determination in fuzzy, amorphous regions. It is worth noting that we focused on these voxel-rather than lesion-based measures to assess the precise delineation of WML boundaries, which are not directly assessed with lesion-based measures^52^.

Despite improvement, several limitations remain. As discussed in the literature, WML segmentation of age-related, mild loads is a challenging problem with the low detection rates in the 30% range as opposed to the > 80% range seen with large WML loads (> 15 mL). In this study, the average Dice index was lower than 0.5 for all automatic methods, in line with the current state of the literature^25,29,45,50,51^ and not as promising as segmentation of the typically large multiple sclerosis (MS) lesions reported in the literature^22^. The main cause of low indices is likely missed segmentations in parts of the brain that contain high density white matter fibers, with small intensity differences between the white matter lesions and background normal white matter fibers. When detecting WML visually, neuroradiologist rely on the signal intensity gradient (difference) between the WML and the surrounding normal white matters. This is easily done qualitatively when the gradient is high between the hyperintense WML and the areas in the brain with low density white matter fibres with relatively low intensity background. Areas of the brain with high density white matter fibres have a relatively higher intensity than the rest of the brain which reduces the gradient of signal intensity between the high-density white matter and the hyperintensity WML. Nonetheless, an experienced neuroradiologist can still detect these WML despite this slight challenge to the visual segmentation by adjusting the window width and window length when analysing the images. Areas with such fibers are the corona radiata and centrum semiovale, which contain major neural networks in the brain such as the cortico-spinal tracts. Consistent with this finding, the automatic methods struggle to segment WML in such low contrast and fuzzier white matter areas. Similarly, the per-voxel metrics were low (PPV, TPR, and FPR) for mild WML burden, although the proposed method is superior to the other methods for most WML loads. Overall volumes corresponded well with ground truth, indicating that while individual voxels may be inappropriately assigned, the overall segmented sizes of WML are accurate with the proposed method. Detection was also limited by the 5 mm slice thickness and 1 mm inter-slice gaps, which can reduce contrast or miss lesions within gaps^53^, respectively. Imaging on higher resolution MRI scanners may yield higher detection rates and better differentiation of false from true lesions.

Detecting outliers is a critical problem in defining WML since noise, incomplete skull stripping, and other artefacts often mimic WML, with the potential for false diagnoses, undue distress, unnecessary follow-up testing, or even unwarranted treatment. Previous methods have focused on identifying the features of healthy normal brain tissue to identify WML, whereas the proposed approach utilizes characteristics inherent in outliers to classify them as WML or non-WML, using intensity voxel standardization together with cluster-based GLCM texture feature extraction to enhance and contrast these features with normal tissue. Moreover, the LOF algorithm further curates lesion boundaries by adaptively comparing voxel intensities with their local neighbourhood, which allows for more precise WML segmentation in fuzzy areas of low contrast (see Fig. 4g).

By reducing the burden of false diagnoses from WML segmentation, the proposed method can serve as a more reliable and quantitative WML measurement tool for clinical practice. For example, tools to detect and quantify the burden and trajectories of WML early in disease may enhance the capability for early diagnosis of leukoencephalopathies, quantify WM changes related to headache (e.g., migraine), and find patterns that might distinguish vascular dementia from other forms of dementia. Additionally, having the segmentation results in selected patients can be useful as it provides a more accurate quantification that may have a significant role in deciding whether a particular intervention should be continued, stopped or changed. Furthermore, if the outlining is accurately completed by automatic segmentation, where clinicians only need to cross-check or make subtle adjustments, the threshold to performing accurate segmentation for research or clinical trials will be lowered. We also anticipate that segmentation may adopt an important future clinical role for quantitative analysis of disease, which allows for lesion volume tracking to assess disease progression and assessment of response to treatment both for clinically approved and novel pharmaceuticals. Identification of lesion patterns and distributions may also serve as biomarkers for diseases and disease sub-types.

Deep learning is well-recognized as a promising technique for computer aided diagnosis. However, the training process of deep learning typically requires a large amount of WML-related, copiously annotated image data for sufficient training without overfitting to selected populations, coupled with computationally intensive training calculations. To the best of our knowledge, such datasets capturing the broad scope of WML image data are not yet available^38^, particularly for the subtler lesions of early age-related diseases targeted here. Although pre-trained CNN models such as VGG 16, Inception V3, and ResNet 50 are useful starting points to pilot such algorithms, most of the trained image data in these models are not related to the medical diagnosis, especially WML imaging. Classical image processing algorithms such as the random forest algorithm have the advantage of operating as fully unsupervised techniques that do not require this breadth of training. We chose the random forest method as it has been verified as the best performer specifically for medical segmentation of WML among ten other classification algorithms^54^. Another common limitation of supervised learning algorithms is the requirement to retrain detection models constructed by non-invariant features when MR scanner acquisition settings change^47^. However, in our proposed framework, this retraining is not required because the MR voxel intensities are corrected and standardised during the pre-processing stage using the landmark based intensity standardization method^55^. A drawback is that the constructed model is specific to WML, and hence for brain lesions that predominate in gray matter such as many neurodegenerative diseases, the model’s settings would need to be retrained.

## Conclusion

We have proposed a method designed to effectively reduce the FP of WML for accurate WML segmentation, tailored to subtle lesions that are characteristic of early age-related neurological brain disorders. As a future extension, the proposed method can be applied to longitudinal population studies for further validation and identification of the earliest accurate detection of the onset for many white matter disorders, interrogating whether and what types of subtle abnormalities detected as WML go on to declare themselves as unequivocal pathology. By enabling comparison of texture of WML and MR intensity across different time points, the evolution of white matter disease can be modeled from its onset and better correlated with genetic, environmental and therapeutic measures. Also, it will be interesting to further extend and apply this method to help differentiate different types of WML that otherwise share similar appearance and signal characteristics through texture differences, helping to unlock the underlying classifications and associated etiologies of WML in neurological diseases.

## Materials and methods

### Subjects, imaging, and WML loads

The proposed WML segmentation and false positive elimination method was evaluated using a subset of cranial MR images obtained from a clinical study of 121 subjects older than 35 years with cardiovascular risk factors on the protective effect of palm vitamin E tocotrienols on brain white matter^15^, scanned using the brain MR imaging protocol for T1-Weighted (T1-W) and FLAIR sequences listed in Table 3. Informed consent was obtained from all volunteers and the study was approved by the Research Ethics Committee for human studies of Universiti Sains Malaysia (http://www.crp.kk.usm.my/pages.jepem.htm), and all methods were carried out in accordance with relevant guidelines and regulations. From this study population, we randomly selected 42 subjects to perform detailed annotations on their baseline MR images. This number provided for 10 training and at least 30 test subjects, which allowed for reasonable assumptions of normality in the sampling distribution of the test cases and as a practical case load for meticulous ground truth annotations by the neuroradiologists.

**Table 3:** MRI protocol specification for the FLAIR and T1-W sequence obtained from a 1.5 T Signa HDx GE Scanner and detailed information about the use of MRI datasets for training and testing.

Sequence	FLAIR	T1-W
**MRI protocol**		
Orientation	2D-Axial	2D-Axial
Image dimension (px)	512 × 512	512 × 512
Voxel size (mm)	0.4297 × 0.4297 × 5	0.4297 × 0.4297 × 5
Intersection gap (mm)	1	1
Repetition time (ms)	8002 ± 0	490.70 ± 29.39
Echo time (ms)	126.21 ± 2.30	13.30 ± 0.46
Inversion time (ms)	2000 ± 0	–

Dataset	Training (FLAIR)	Testing (FLAIR)
**MRI data set**		
Subject's gender (Male/Female)	10 (6/4)	32(13/19)
Subject's age	56.40 ± 10.71	56.38 ± 6.46
Slices of each subject	20.455 ± 6.905	21.55 ± 4.139
Confirmed WML slices/total slices	92/225	279/711
Confirmed WML counts	533	1569
“WML”/“Non-WML” annotation counts	533/3924	–
WML loads (mL) of each subject	5.040 ± 7.05	7.480 ± 23.82
Mild WML loads (mL)/Subjects	1.459 ± 1.4730/7	1.639 ± 0.9936/25
Moderate WML loads (mL)/Subjects	5.909 ± 0.3206/2	8.855 ± 2.147/ 5
Severe WML loads (mL)/Subjects	23.951/1	77.11 ± 83.62/2

Repetition time, Echo time, Inversion time, Age, Slices of each subject, and WML loads are presented as mean ± standard deviation. ms, mm, and mL represent for milliseconds, millimeters, and milliliters, respectively.

We further broke down the target study population by lesion load to obtain a larger proportion of those with low loads while including some with higher loads to ensure applicability of the method to the full range of WML loads. To obtain an approximation of the total lesion load for each subject in the original cohort, we used the trimmed mean outlier method^23^. Next, we randomly selected 32 subjects with less than 5 mL loads and randomly partitioned 7 for training and 25 for testing. For medium loads (5–15 mL), we randomly selected another 10 samples, partitioning 3 for training and 7 for testing. Last, we selected 3 subjects (1 for training, 2 for testing) with high lesion loads (greater than 15 mL) as positive controls to ensure that our segmentation could generalize to the more common and less challenging case of large lesions.

The training data set for the random forest classifier was validated by tenfold cross-validation of 10 subjects with a total of 92 2D FLAIR planes comprising 533 individual white matter lesions in patches of different sizes and 50.43 ml total lesion load (mean 5.04 ± 7.05 mL; see Table 3). For testing, a total of 32 subjects comprising a total of 279 2D FLAIR planes with a total count of 1569 individual white matter lesions and a cumulative WML volume of 239.48 mL (mean 7.48 ± 23.82 mL).

### Ground truth and training dataset preparation protocol

Two neuroradiologists prepared the ground truth annotations using the publicly available software package MIPAV (Medical Image Processing, Analysis and Visualization), provided by the National Institutes of Health (NIH), which includes a tool for manual delineation assisted by a level set segmentation tool as a method of modeling curve evolution based on contour shape. For each lesion, the neuroradiologist painted hyperintense pixels as seed points, which the level-set tool expands to include similar hyperintensities in the immediately adjacent, neighboring pixels. This assistive segmentation both speeds the annotation process and captures pixels that are difficult to annotate accurately with standard painting tools. The level-set threshold can be titrated to limit its aggressiveness, especially in areas with obscured borders. The annotation boundaries are composed of splines that allow further fine-tuning through manual, free-form movement to ensure close alignment with the underlying lesion. When considering the extent of lesion boundaries, the neuroradiologist incorporated knowledge from the adjacent slices above and below each given slice and adopted a liberal annotation strategy that included spatially heterogeneous patterns such as those found in deep WML and periventricular WML as well as intermediate phases of hyperintensity surrounding many of the bright hyperintensities, fainter than the core but brighter than the surrounding normal tissue^56,57^. One neuroradiologist first generated a complete set of annotations for all subjects, followed by verification of each annotation by a second neuroradiologist. When the neuroradiologists disagreed on a lesion, this second neuroradiologist would generate an independent annotation. Using the Intra-Class Correlation metric on lesion volume, the two neuroradiologists demonstrated consistent results across annotations (ICC = 0.9950, 95% CI: 0.9897, 0.9976). Details and comparison of their agreement measures can be viewed in Supplementary Material Tables S1–S3.

Next, we prepared the training dataset for WML detection and segmentation by applying TMOD^23^ to obtain separate sets of true and false WML, both necessary to train the classifier. We used the annotations drawn by the neuroradiologist as the true WML training set, labelled as "Lesions," and the remaining TMOD output lesions that did not match the neuroradiologist's annotations as the false WML, "Non-lesion" set. Finally, all WML and non-WML annotations were converted into binary masks for the feature extraction in the next process. This approach reduces the neurologist's workload while simultaneously making the segmentation results more realistic for the RF classifier.

### Image pre-processing

In the WML segmentation framework, N3 inhomogeneity correction, skull stripping, intensity standardisation, and image registration are indispensable preprocessing steps to accurate WML detection and segmentation (see Fig. 1a). In our segmentation work, we first perform intra-subject co-registration of each 2D T1-weighted image to the corresponding 2D FLAIR image. This registration method is to use the built-in function of automatic multi-modal medical image registration provided in the MATLAB 2014a (Mathworks Inc., USA) image processing toolbox. It uses intensity-based image registration to automatically align two magnetic resonance (MRI) images to a common coordinate system. Next, we implemented the N3 inhomogeneity correction method suggested by Sled et al.^58^ to eliminate the MRI artefacts caused by receiver coil sensitivity variation during the MRI scanning process^59^ on co-registered T1-W and FLAIR sequence images, which show a gradient effect on the image from side to side and inhibit brain segmentation. After attenuating these artifacts, we proceeded to segment the brain into white matter, gray matter, and cerebrospinal fluid by using a fuzzy C-means clustering algorithm^60^. These regions can be used in the next step to further identify and eliminate false positives as reported in our previous study^23^.

Next, we used the level-set algorithm introduced by Zhuang et al.^61^ to strip the skull from brain tissue. Compared with other skull stripping algorithms using morphological operations, the level-set approach obtained a superior segmentation when incorporating the patient’s age (obtained from DICOM metadata) as the key criterion to terminate the segmentation evolution for skull stripping. Co-registered T1-W is the input for the skull stripping process because neuroradiologists use T1-W as the best MRI sequence to show brain tissue structure. Next, the skull stripped T1-W images were used as a mask to extract the brain in the corresponding FLAIR images. MRI intensity standardisation was applied using the process that we described previously^55^ because the voxel intensity read from MRI shows large variations between and within each scan due to the limitation of MRI instrumentation, which would otherwise preclude texture and brightness comparability between images. The output of standardised FLAIR MRI (see Fig. 1c) was used to perform the feature extraction later.

For WML segmentation, the method based on boxplots and trimmed mean computation described in our previous study^23^ was used for automated WML segmentation and extended here to avoid subtle false positives. First, we used the trimmed mean method to estimate the normal brain intensity distribution in each 2D image slice. Next, we constructed boxplots of each distribution, where extreme outliers identified the presence of WML in each slice and defined the threshold parameters for WML segmentation (see Fig. 1b). By using boxplots specific to each 2D slice, we thus adapt to local outliers while maintaining a global context to minimize subtle false positives.

### GLCM embedded clustering strategy for WML detection and false positive removal

Among the candidates for detected WML, false positives remained such as hyperintensity voxels, seen especially in mild WML loads, image flow artefacts, incomplete skull stripping, and the non-specific (poor signal-to-noise ratio) caused by inaccurate intensity thresholding in WML detection^62^. However, current TMOD methods^23^ have not been shown to delineate these hyperintensity types for false positive removal. In this study, we propose a method based on the standardised intensity^55^ of WML candidates and their textures to enhance the features to identify and classify WML by using random forests, substantially reducing false positives.

**Figure 6:**
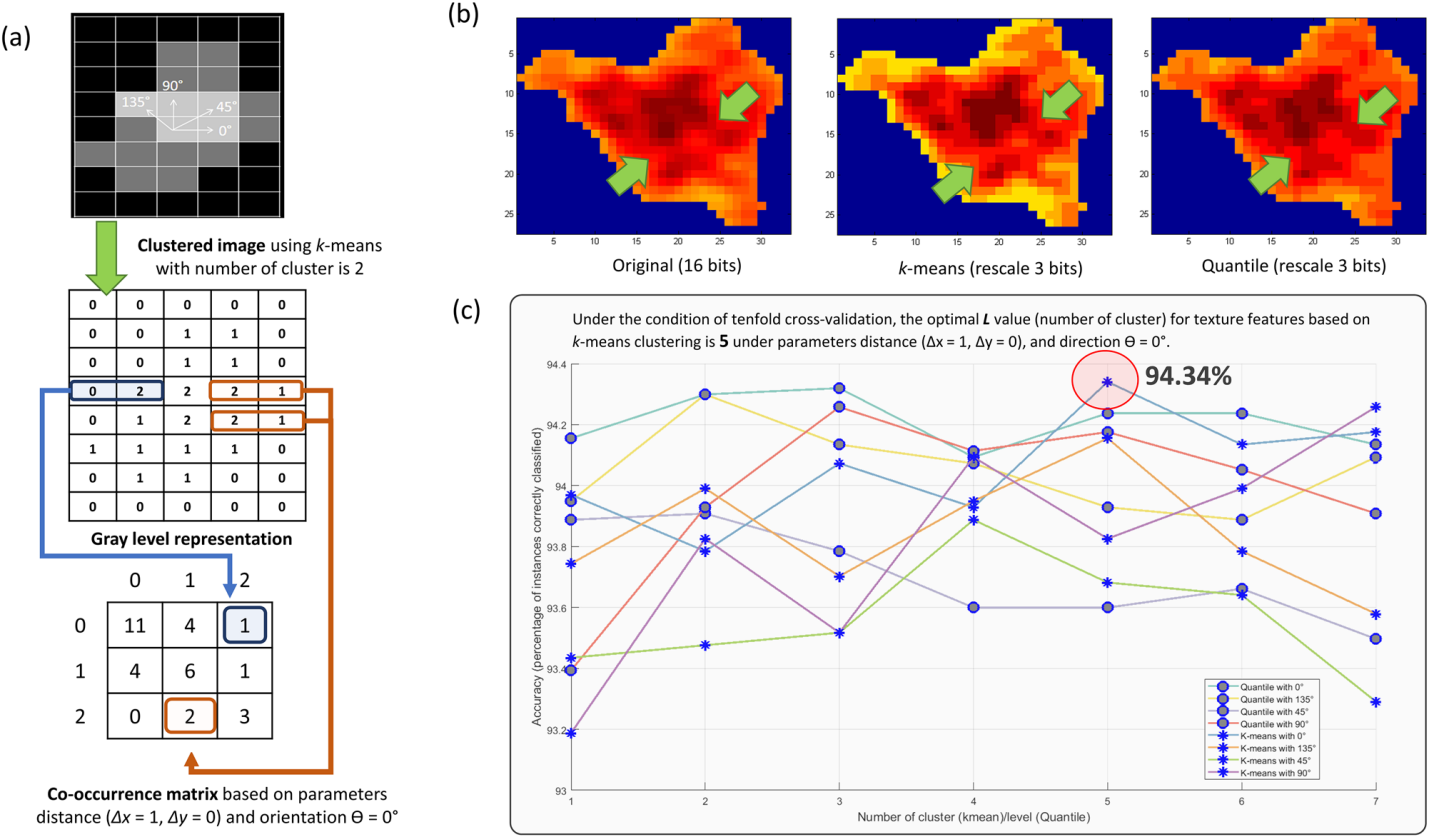
(**a**) Geometrical relationship of GLCM computed based on distance *d* = 1 and four directions, where *Ɵ* = 0°, 45° 90°, and 135°. (**b**) The result of texture visualization after rescaling the intensity information from 16 to 3 bits through *k*-means and the quantile method. (**c**) The optimal *k* value of *k*-means and the quantile method determined in the tenfold cross validation experiment using the training dataset.

To identify and eliminate FP, we first apply the Random Forest algorithm, known for its suitability to high-dimensional and multi-classification problems in medical images^54,63^.One of the advantages of this algorithm is that it can classify without requiring feature scaling, a time-consuming and error-prone step. Two important features extracted from the WML image patch serve as input to the Random Forest: (1) the intensity histogram feature (see Table 4), calculated based on WML candidate intensities obtained from WML image patches, and (2) the texture feature from the GLCM embedded clustering strategy that we propose here, where we embedded *k*-means clustering in a gray level co-occurrence matrix (GLCM) calculation. In Fig. 6b, we compared the texture visualization between the original WML candidate image and the reconstructed image determined by *k*-means versus the quantile method (the existing linear scaling method), which demonstrated that *k*-means can recapitulate the detailed texture of the original image with greater fidelity, as highlighted by the green arrow. This shows that the clustering method can effectively scale the original image from 16 to 3 bits, while still retaining the global content. In addition, the *k*-means clustering algorithm has been successfully and widely used in various medical image segmentation applications such as WML segmentation^64,65^, brain lesion segmentation^66^, brain segmentation^67^, corpus callosum segmentation^68^, and brain tumour segmentation^69,70^. In our implementation, *k*-means was used to accurately cluster the intensity data of WML texture structure, so that the feature data in the next feature extraction process was more accurate in the GLCM calculation (see Fig. 1d). Therefore, the intensity of each image patch will be clustered as the input to the GLCM calculation.

**Table 4:** Discrete probabilities $p({g}_{i})$ of gray levels ${g}_{i}$, with $i=0,\dots \dots N-1(L=8192)$, in a standardised image.

Features of intensity histogram	Definition
Mean, µ	$\sum_{i=0}^{N-1}{g}_{i}P({g}_{i})$
Variance, σ^2^	$\sum_{i=0}^{N-1}{({g}_{i}-\mu )}^{2}P({g}_{i})$
Skewness	$\frac{\sum_{i=0}^{N-1}{{(g}_{i}-\mu )}^{3}P({g}_{i})}{{\sigma }^{3/2}}$
Kurtosis	$\frac{\sum_{i=0}^{N-1}{{(g}_{i}-\mu )}^{4}P({g}_{i})}{{\sigma }^{2}}$
Energy	$\sum_{i=0}^{N-1}{P}^{2}({g}_{i})$
Entropy	$\sum_{i=0}^{N-1}P({g}_{i}){log}_{2}P({g}_{i})$

GLCM is a method of describing and digitizing image texture by finding the frequency of pixel pairs that appear in various spatial relationships (such as distance and direction) in an image. These frequencies was used to construct the gray-level spatial correlation matrix, also known as a co-occurrence matrix (CM)^71^, where the element of the co-occurrence matrix for a given clustered WML image patch *N* × *M* with *L* clustered grey levels can be defined as Eq. 1. Subsequently, the GLCM was constructed from each of the elements in the matrix of probabilities that pairs of pixels occurred in a given spatial relationship. For instance, *Prob(x,y|∆x*,*∆y)* (see Eq. 2), which was computed based on the distance *d*(*∆x*, *∆y*) of two neighboring voxels in the clustered intensity image that co-occur at a given orientation (0°, 45°, 90°, and 135°).

1 $$CM=\left\{\begin{array}{ll} 1&\,if\,f\left(x,y\right)=n\,AND\,f\left(x-\Delta x, y-\Delta y\right)=m \\ 0 &else where\end{array}\right.$$

2 $$Prob\left(x,y|\Delta x,\Delta y\right)= \frac{1}{\left(N-\Delta x\right)(M-\Delta y)}\sum_{y=1}^{M-\Delta y}\sum_{x=1}^{N-\Delta x}CM$$

Therefore, each element *Prob(x,y|∆x*,*∆y)* represents the relative frequency based on their neighbourhood relationship as illustrated in Fig. 6a. Subsequently, texture features including contrast, energy, correlation, and homogeneity features were calculated from the GLCM (see Table 5).

**Table 5:** Discrete probabilities $P(i,j)$ of gray level co-occurrence matrix for distance *d* is (1,1), with $N$ is the number of clusters used in an image of a lesion.

Gray level co-occurrence matrix (GLCM) texture features	Definition
Contrast	$\sum_{i=0}^{N}\sum_{j=0}^{N}{\left(i-j\right)}^{2}P\left(i,j\right)$
Energy	$\sum_{i=0}^{N}\sum_{j=0}^{N}{P}^{2}\left(i,j\right)$
Correlation	$\sum_{i=0}^{N}\sum_{j=0}^{N}\frac{\left\{i\times j\right\}\times P\left(i,j\right)-{\mu }_{x}{\mu }_{y}}{{\sigma }_{x}{\sigma }_{y}}$
Homogeneity	$\sum_{i=0}^{N}\sum_{j=0}^{N}\frac{P(i,j)}{1+|i-j|}$

${\mu }_{x},{\mu }_{y},{\sigma }_{x}\text{, and }{\sigma }_{y}$ are the mean and standard deviations of ${P}_{x}\text{and }{P}_{y}$. ${P}_{x}(i)$ is the *i*-th entry in the marginal-probability matrix obtained by summing the rows of *P*(*i, j*): ${P}_{x}\left(i\right)=\sum_{i=0}^{N-1}P(i,j) \text{ and }{P}_{y}\left(j\right)=\sum_{j=0}^{N-1}P(i,j)\text{; }{\mu }_{x}=\sum_{i=0}^{N-1}i{P}_{x}(i){ {\text{ and }} \mu }_{y}=\sum_{j=0}^{N-1}i{P}_{y}(j)$.

$${\sigma }_{x}=\sum_{i=0}^{N-1}{(P}_{x}\left(i\right)-{\mu }_{x}\left(i\right){{)}^{2} {\text{ and }} \sigma }_{y}=\sum_{j=0}^{N-1}({P}_{y}\left(j\right)-{\mu }_{y}(j){)}^{2}$$.

Three parameters need to be considered to compose the best GLCM texture and intensity histogram feature set: (1) the number of clusters when constructing cluster images, (2) the parameters needed to identify pixel pairs when constructing GLCM, which are the distance (∆x = 1, ∆y = 0) and (3) the orientation (0°, 45°, 90°, 135°). The distance (∆x = 1, ∆y = 0) was fixed because the size of patch images was relatively small. In our study, the optimal parameter for the number of clusters and direction was validated on the training dataset consisting of 533 “WML” and 3924 “Non-WML” using Random Forest in a tenfold stratified cross-validation experiment using WEKA 3.6^72^ (see Fig. 6c). In brief, this process produced 10 equally sized sets for training and testing to generate a random forest classifier. In the first iteration, 90% of the cases are used for training, with the remaining 10% of the cases used for testing to obtain a performance metric such as accuracy. The same process was repeated nine times so that each case was used once in a test group, providing a sufficient ratio of training to testing and true to false lesions. We took the average accuracy rate from the ten iterations as the final accuracy of the random forest classifier. Accuracy of WML classified based on each ***L***, the number of cluster (range from 1 to 7), and *G* level processes using the *k*-means algorithm, and the quantile method (the same method used to rescale the intensity before GLCM calculation), respectively, were compared with the four orientations at the distance (*∆x* = 1, *∆y* = 0). The optimal ***L*** (number of cluster) for *k*-means cluster-based texture features was 5 for the orientation Ɵ = 0°, with a highest accuracy of 94.34% validated in the test dataset. Similarly, the optimal random forest parameters for number of trees, *T* = 25, and tree depth, *D* = 25, have been validated based tenfold cross validation using optimal cluster-based texture features. Additional description about optimum parameters applied in Random Forest classifier is provided in the Supplementary Material, Sect. 4, and our *k*-means implementation in Supplementary Material, Sect. 5.

### Defining WML region boundary using local outlier factor

The boundaries of the detected WML regions were segmented and defined using the Local Outlier Factor (LOF) algorithm originally introduced by Breunig et al.^44^. The Trimmed Mean Outlier method^23^ previously used to segment WML may be difficult to accurately define the boundary of diffuse WML. Furthermore, the spatial distribution of WML is varied since it can be found in a wide variety of brain tissues and thus subjected to artifacts from surrounding voxels. Knowledge of the neighbour voxel intensity is crucial to identify a reasonable boundary of each WML regionally and adaptively.

We proposed the local outlier factor (LOF) to overcome this limitation (see Fig. 1e), a method that has not yet been applied to medical image segmentation to the best of our knowledge. First, each voxel was classified as normal or outlier on identified WML image patches to measure the extent to which each outlier deviates from its local neighbourhood. The image intensity features in the WML training set outlined by the neuroradiologist was used to construct the intensity gray level model. The LOFs were constructed in the following steps:

1. Identify the *k*_α_-neighbours by calculating distances *d*(*o*_α_, *p*_α_) in between each *o*_α_ voxel intensity and *p*_α_ in training data for each voxel, where *k*_α_ is a natural number used to build the number of neighbours. Obtain the *k*_α_-distance neighbourhood by obtaining the first *k*_α_-distance nearest to *o*_α_ as defined in Eq. (3)
  3 $${k}_{\alpha }\text{-neighours =}d\left({o}_{\alpha },{p}_{\alpha }\right)\le {k}_{\alpha }-\text{distance(o)}$$
2. Reachability distances are defined in Eq. (4), where *β* is test data, *d*(*p*_*β*_,o) is distance between *p*_*β*_ from *k*_*β*_-neighbours and *o ∈ β*
  4 $${reach-dist}_{k}\left(p,o\right)=max\left\{k-\mathrm{distance}\left(o\right),d({p}_{\beta },o)\right\}$$
3. Local reachability distances are defined in Eq. (5), which calculate the distance between each voxel intensity *o ∈ β* and all neighbours *p*_α_* ∈ MinPts*_*α*_(*o*) of *k*_*α*_-neighbours, where *MinPts*_*α*_ is a minimum number of voxel intensities in the neighbourhood of a voxel intensity *o*.
  5 $$lrd_{{MinPts_{\alpha } }} \left( o \right) = {\raise0.7ex\hbox{$1$} \!\mathord{\left/ {\vphantom {1 {\left( {\frac{{\sum _{{p \in MinPts_{\alpha } (o)}} reach - dist_{{MinPts_{\alpha } }} (o,p)}}{{N_{{MinPts_{\alpha } (o)}} }}} \right)}}}\right.\kern-\nulldelimiterspace} \!\lower0.7ex\hbox{${\left( {\frac{{\sum _{{p \in MinPts_{\alpha } (o)}} reach - dist_{{MinPts_{\alpha } }} (o,p)}}{{N_{{MinPts_{\alpha } (o)}} }}} \right)}$}}$$
4. Calculate Local Outlier Factor (Eq. 6) for each voxel intensity *o ∈ β.*
  6 $${LOF}_{{MinPts}_{\alpha }}\left(o\right)=\frac{{\sum }_{p\epsilon MinPts(o)}\frac{{lrd}_{{MinPts}_{\alpha }}(p)}{{lrd}_{{MinPts}_{\alpha }}(o)}}{{n}_{{MinPts}_{\alpha }}(o)}$$

Detailed analysis of the LOF properties and theorem can be found in the study conducted by Breunig et al.^44^. The output generated with the LOF algorithm was considered the final WML at the end of the framework as demonstrated in Fig. 1e.

### Statistical analysis

The Spearman correlation coefficient and Intra-Class Correlation coefficient (ICC) tests were employed to measure the correlation and agreement, respectively. A Pearson correlation value of 1 indicates the best correlation, and a *p-*value of < 0.05 is considered statistically significant. For testing the agreement, ICC and its corresponding 95% confidence interval (CI) were calculated to determine the inter-observer agreement of the WML load detected by automated segmentation with the manual delineations. In addition, we used a paired t-test to compare the evaluation metrics between methods and employed Bonferroni correction to correct the significance level for multiple comparisons. All statistical analyses were performed in MATLAB 2014a (Mathworks Inc., USA) and its statistical toolbox.

### Consent for publication

All authors of this work agreed to publish with Nature Scientific Reports.

## Supplementary Information

Supplementary Information.

## Data Availability

Due to sample information protection, patient privacy protection and medical institutional data regulatory policies, the data for the development and verification of the proposed method are not publicly available, but Prof Emeritus Dr. Yuen Kah Hay (khyuen@usm.my) can be contacted directly.

## Code availability

The source code for the methods and their optimum training models are available at https://github.com/kokhaur/WML-Segmentation-False-Positive-Detection.

## References

[1] Yamauchi H, Fukuda H, Oyanagi C (2002). Significance of white matter high intensity lesions as a predictor of stroke from arteriolosclerosis. J. Neurol. Neurosurg. Psychiatry.

[2] Debette S, Markus HS (2010). The clinical importance of white matter hyperintensities on brain magnetic resonance imaging: Systematic review and meta-analysis. BMJ.

[3] Cavalieri M (2010). Vascular dementia and Alzheimer's disease: Are we in a dead-end road?. Neurodegener. Dis..

[4] Park MH (2010). Vascular risk factors and the effect of white matter lesions on extrapyramidal signs in Alzheimer's disease. Int. Psychogeriatr..

[5] Kawata Y (2010). Computer-aided evaluation method of white matter hyperintensities related to subcortical vascular dementia based on magnetic resonance imaging. Comput. Med. Imaging Graph..

[6] Alber J (2019). White matter hyperintensities in vascular contributions to cognitive impairment and dementia (VCID): Knowledge gaps and opportunities. Alzheimer's Dementia.

[7] Jonsson M (2010). Cerebrospinal fluid biomarkers of white matter lesions: Cross-sectional results from the LADIS study. Eur J Neurol.

[8] Launer LJ (2004). Epidemiology of white matter lesions. Top. Magn. Reson. Imaging.

[9] O'Sullivan M (2008). Leukoaraiosis. Pract. Neurol..

[10] Silbert LC (2008). Impact of white matter hyperintensity volume progression on rate of cognitive and motor decline. Neurology.

[11] Pinter D (2017). Impact of small vessel disease in the brain on gait and balance. Sci. Rep..

[12] Zheng JJJ (2011). Impact of white matter lesions on physical functioning and fall risk in older people. Stroke.

[13] Chutinet A, Rost NS (2014). White matter disease as a biomarker for long-term cerebrovascular disease and dementia. Curr. Treat. Opt. Cardiovasc. Med..

[14] Schmidt R (2003). Progression of cerebral white matter lesions: 6-year results of the Austrian Stroke Prevention Study. Lancet.

[15] Gopalan Y (2014). Clinical investigation of the protective effects of palm vitamin E tocotrienols on brain white matter. Stroke.

[16] Fox RJ (2011). Advanced MRI in multiple sclerosis: Current status and future challenges. Neurol. Clin..

[17] Enzinger C (2015). Nonconventional MRI and microstructural cerebral changes in multiple sclerosis. Nat. Rev. Neurol..

[18] Scheltens P (1993). A semiquantative rating scale for the assessment of signal hyperintensities on magnetic resonance imaging. J. Neurol. Sci..

[19] Wahlund LO (2001). A new rating scale for age-related white matter changes applicable to MRI and CT. Stroke.

[20] Fazekas F, Chawluk JB, Alavi A (1987). MR signal abnormalities at 1.5 T in Alzheimer's dementia and normal aging. Am. J. Roentgenol..

[21] Enzinger C (2007). Progression of cerebral white matter lesions: Clinical and radiological considerations. J. Neurol. Sci..

[22] Caligiuri ME (2015). Automatic detection of white matter hyperintensities in healthy aging and pathology using magnetic resonance imaging: A review. Neuroinformatics.

[23] Ong KH (2012). Automatic white matter lesion segmentation using an adaptive outlier detection method. Magn. Reson. Imaging.

[24] Styner M (2008). 3D Segmentation in the Clinic: A grand challenge II: MS lesion segmentation. MIDAS J..

[25] Roura E (2015). A toolbox for multiple sclerosis lesion segmentation. Neuroradiology.

[26] Schmidt P (2017). Bayesian Inference for Structured Additive Regression Models for Large-Scale Problems with Applications to Medical Imaging.

[27] Schmidt P (2019). Automated segmentation of changes in FLAIR-hyperintense white matter lesions in multiple sclerosis on serial magnetic resonance imaging. Neuroimage.

[28] Schmidt P (2012). An automated tool for detection of FLAIR-hyperintense white-matter lesions in Multiple Sclerosis. Neuroimage.

[29] Ribaldi F (2021). Accuracy and reproducibility of automated white matter hyperintensities segmentation with lesion segmentation tool: A European multi-site 3T study. Magn. Reson. Imaging.

[30] Vanderbecq Q (2020). Comparison and validation of seven white matter hyperintensities segmentation software in elderly patients. Neuroimage.

[31] Wu M (2006). A fully automated method for quantifying and localizing white matter hyperintensities on MR images. Psychiatry Res..

[32] Udupa JK (1997). Multiple sclerosis lesion quantification using fuzzy-connectedness principles. IEEE Trans. Med. Imaging.

[33] Udupa JK, Saha PK, Lotufo RA (2002). Relative fuzzy connectedness and object definition: Theory, algorithms, and applications in image segmentation. IEEE Trans. Pattern Anal. Mach. Intell..

[34] de Boer R (2009). White matter lesion extension to automatic brain tissue segmentation on MRI. Neuroimage.

[35] Guizard N (2015). Rotation-invariant multi-contrast non-local means for MS lesion segmentation. Neuroimage.

[36] Sudre CH (2015). Bayesian model selection for pathological neuroimaging data applied to white matter lesion segmentation. IEEE Trans. Med. Imaging.

[37] Ghafoorian M (2017). Location sensitive deep convolutional neural networks for segmentation of white matter hyperintensities. Sci. Rep..

[38] Ding T (2020). An improved algorithm of white matter hyperintensity detection in elderly adults. Neuroimage.

[39] Wen W (2009). White matter hyperintensities in the forties: Their prevalence and topography in an epidemiological sample aged 44–48. Hum. Brain Mapp..

[40] Bailey WM (2007). Fast Fluid Attenuated Inversion Recovery (FLAIR) imaging and associated artefacts in Magnetic Resonance Imaging (MRI). Radiography.

[41] Chowdhury MH (2011). Age-related changes in white matter lesions, hippocampal atrophy, and cerebral microbleeds in healthy subjects without major cerebrovascular risk factors. J. Stroke Cerebrovasc. Dis..

[42] Ovbiagele B, Saver JL (2006). Cerebral white matter hyperintensities on MRI: Current concepts and therapeutic implications. Cerebrovasc. Dis..

[43] Yamamoto D (2010). Computer-aided detection of multiple sclerosis lesions in brain magnetic resonance images: False positive reduction scheme consisted of rule-based, level set method, and support vector machine. Comput. Med. Imaging Graph..

[44] Breunig MM (2000). LOF: Identifying density-based local outliers. SIGMOD Rec..

[45] Roura, E. *et al*. *An SPM12 Extension for Multiple Sclerosis Lesion Segmentation*. In *SPIE Medical Imaging* (SPIE, 2016).

[46] Koo TK, Li MY (2016). A guideline of selecting and reporting intraclass correlation coefficients for reliability research. J. Chiropr. Med..

[47] Griffanti L (2016). BIANCA (Brain Intensity AbNormality Classification Algorithm): A new tool for automated segmentation of white matter hyperintensities. Neuroimage.

[48] Roy PK (2015). Automatic white matter lesion segmentation using contrast enhanced FLAIR intensity and Markov Random Field. Comput. Med. Imaging Graph..

[49] Geremia E (2011). Spatial decision forests for MS lesion segmentation in multi-channel magnetic resonance images. Neuroimage.

[50] Valverde S (2017). Improving automated multiple sclerosis lesion segmentation with a cascaded 3D convolutional neural network approach. Neuroimage.

[51] Rachmadi MF (2020). Limited one-time sampling irregularity map (LOTS-IM) for automatic unsupervised assessment of white matter hyperintensities and multiple sclerosis lesions in structural brain magnetic resonance images. Comput. Med. Imaging Graph..

[52] García-Lorenzo D (2013). Review of automatic segmentation methods of multiple sclerosis white matter lesions on conventional magnetic resonance imaging. Med. Image Anal..

[53] Bradley WG, Glenn BJ (1987). The effect of variation in slice thickness and interslice gap on MR lesion detection. AJNR Am. J. Neuroradiol..

[54] Dadar M (2017). Performance comparison of 10 different classification techniques in segmenting white matter hyperintensities in aging. Neuroimage.

[55] Ong KH (2018). White matter lesion intensity standardization using adaptive landmark based brain tissue analysis on FLAIR MR image. Int. J. Adv. Soft Comput. Appl..

[56] Grajauskas LA (2019). MRI-based evaluation of structural degeneration in the ageing brain: Pathophysiology and assessment. Ageing Res. Rev..

[57] Habes M (2018). White matter lesions: Spatial heterogeneity, links to risk factors, cognition, genetics, and atrophy. Neurology.

[58] Sled JG, Zijdenbos AP, Evans AC (1998). A nonparametric method for automatic correction of intensity nonuniformity in mri data. IEEE Trans. Med. Imaging.

[59] Madabhushi A, Udupa JK (2005). Interplay between intensity standardization and inhomogeneity correction in MR image processing. IEEE Trans. Med. Imaging.

[60] Paul, G., *et al*. *A Fuzzy C mean clustering algorithm for automated segmentation of brain MRI*. In *Proceedings of the International Conference on Frontiers of Intelligent Computing: Theory and Applications (FICTA) 2013* (Springer International Publishing, 2014).

[61] Zhuang AH, Valentino DJ, Toga AW (2006). Skull-stripping magnetic resonance brain images using a model-based level set. Neuroimage.

[62] Duan G (2019). Boosting magnetic resonance imaging signal-to-noise ratio using magnetic metamaterials. Commun. Phys..

[63] Criminisi A, Shotton J, Konukoglu E (2011). Decision forests for classification, regression, density estimation, manifold learning and semi-supervised learning. Microsoft Res..

[64] Jaini PS, Deepti SK (2014). Image processing application in the detection of white matter lesions. Int. J. Sci. Res. Dev..

[65] Gwo C-Y, Zhu DC, Zhang R (2019). Brain white matter hyperintensity lesion characterization in T(2) fluid-attenuated inversion recovery magnetic resonance images: Shape, texture, and potential growth. Front. Neurosci..

[66] Juang L-H, Wu M-N (2010). MRI brain lesion image detection based on color-converted K-means clustering segmentation. Measurement.

[67] Liu, J. W. & Guo, L. *Selection of initial parameters of K-means clustering algorithm for MRI brain image segmentation*. In *2015 International Conference on Machine Learning and Cybernetics (ICMLC)* (2015).

[68] Bhalerao, G. V. & Sampathila, N. *K-means clustering approach for segmentation of corpus callosum from brain magnetic resonance images*. In *IEEE 2014 International Conference on Circuits, Communication, Control and Computing (I4C)* (2014).

[69] Vijay, J. & Subhashini, J. An efficient brain tumor detection methodology using K-means clustering algorithm. In *2013 International Conference on Communication and Signal Processing* (2013).

[70] Cabria, I. & Gondra, I. *Automated localization of brain tumors in MRI Using Potential-K-means clustering algorithm*. In *12th IEEE Conference on Computer and Robot Vision (CRV)* (2015).

[71] Jain R, Kasturi R, Schunck BG (1995). Machine Vision.

[72] Kohavi R (1995). A Study of Cross-Validation and Bootstrap for Accuracy Estimation and Model Selection.

