# Supplementary material for: Detection of subtle white matter lesions in MRI through texture feature extraction and boundary delineation using an embedded clustering strategy
Source: Sci Rep. 2022 Mar 15;12:4433. doi: 10.1038/s41598-022-07843-8 (PMC8924181; doi:10.1038/s41598-022-07843-8)
Supplement: Supplementary file 1 — Supplementary Information. [file 41598_2022_7843_MOESM1_ESM.docx]

Supplementary Materials for

Detection of subtle white matter lesions in MRI through texture feature extraction and boundary delineation using an embedded clustering strategy

**Section 1: Qualitative Results**

**The quality of the WML detection and segmentation can be visualised in the following figures in this section. The WML binary results delineated by a neuroradiologist and segmented by the proposed method,** Trimmed Mean Outlier Detection (**TMOD)** suggested by Ong et al.[1]**,** SALEM-LS (SLS) implemented by Roura et al.[2], **and** lesion prediction algorithm (LPA) recently introduced by Schmidt et al.[3]. **were then superimposed on top of intensity standardised FLAIR image. Furthermore, the binary data of white matter and white matter lesions were also used for three-dimensional reconstruction.**


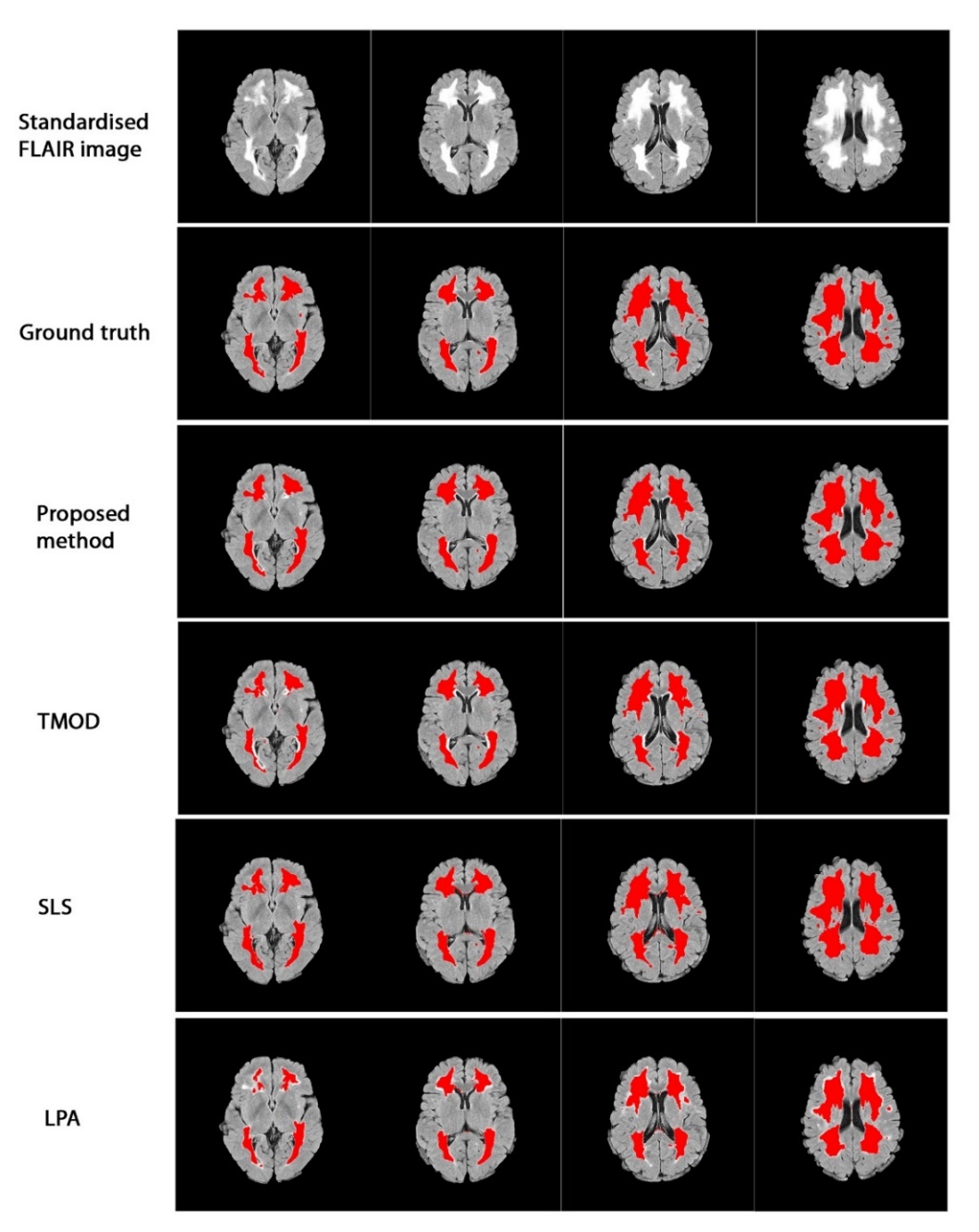


**Fig. S1** Segmented WML is superimposed on top of FLAIR image for the severe case study (> 15mL).


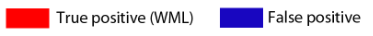


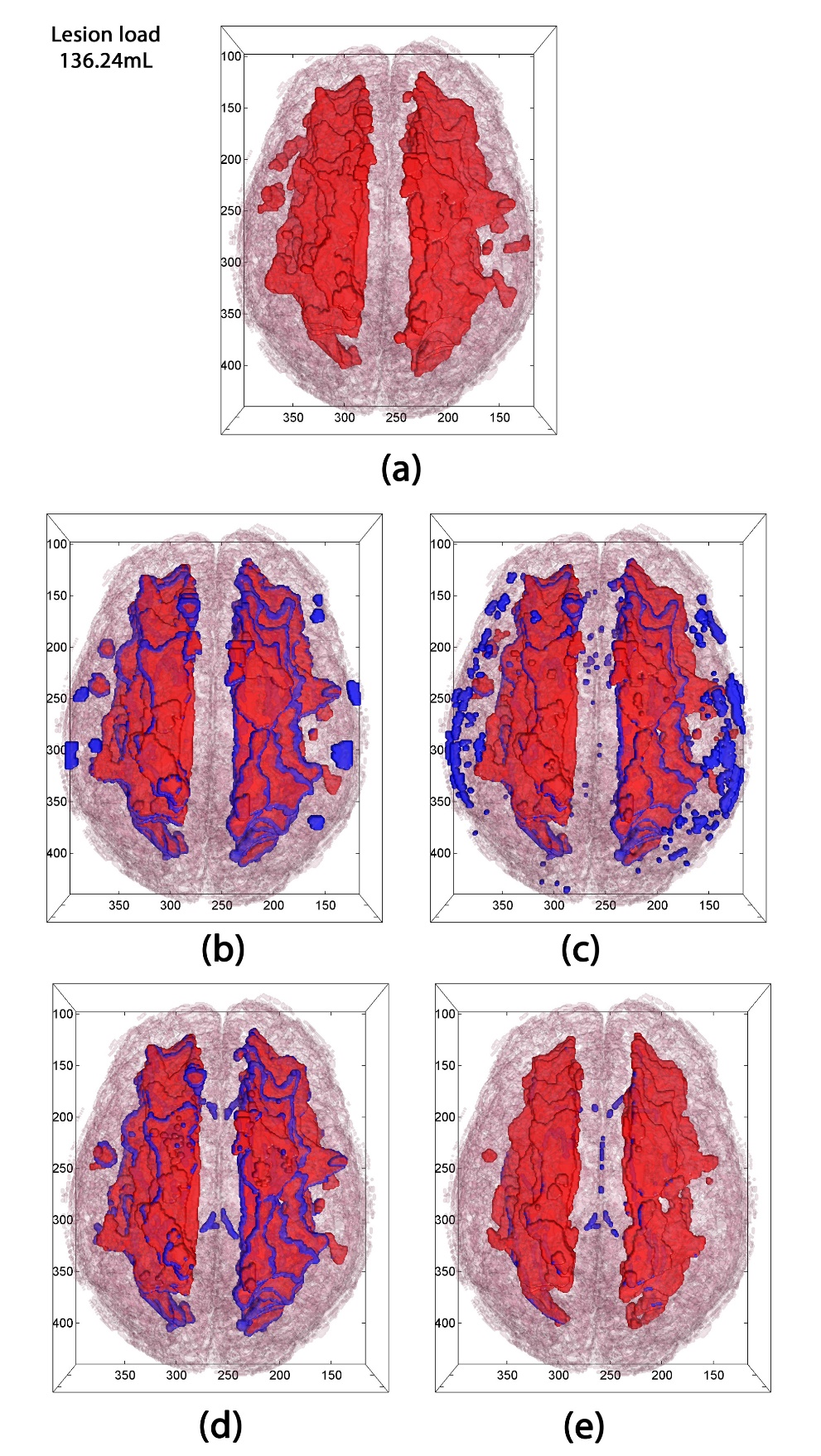


**Fig. S2** Qualitative segmentation results by (a) the neuroradiologist and performance of (b) proposed method, (c) TMOD, (d) SLS, (e) LPA presented in 3D axial view based on the severe case study in Fig. S1.


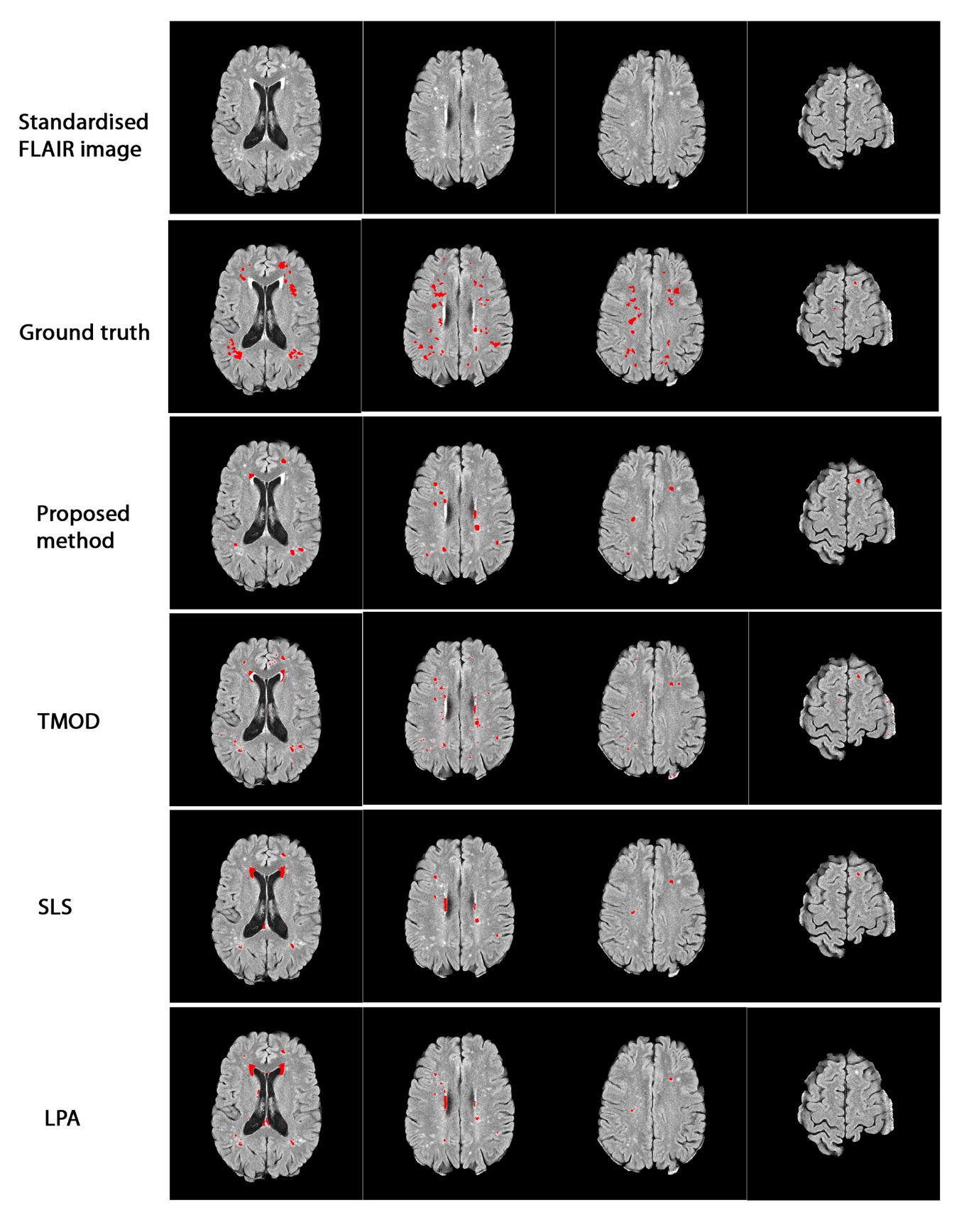


**Fig. S3** Segmented WML is superimposed on top of FLAIR image for the moderate case study (5mL-15mL).


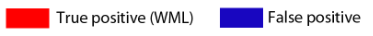


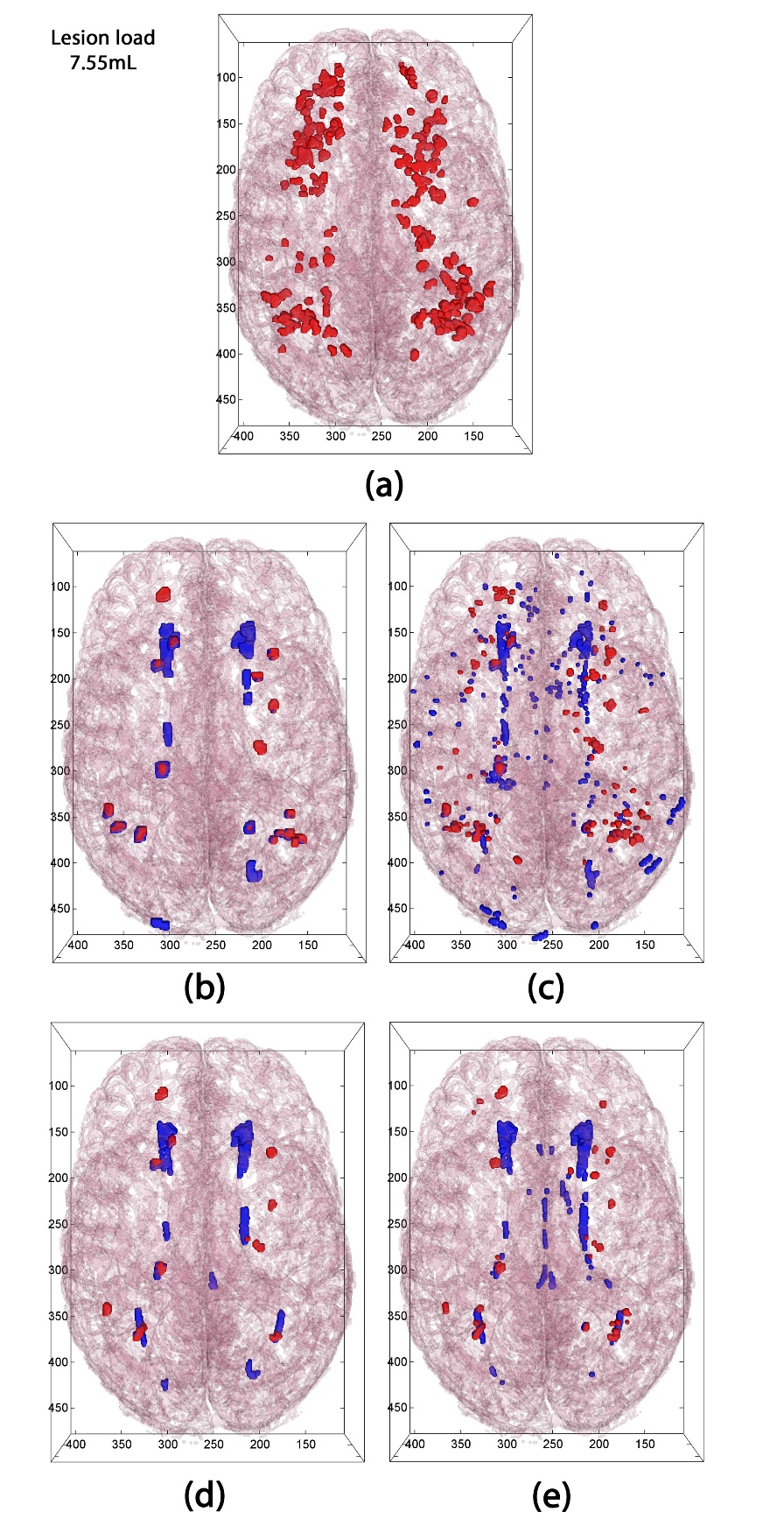


**Fig. S4** Qualitative segmentation results by (a) the neuroradiologist and performance of (b) proposed method, (c) TMOD, (d) SLS, (e) LPA presented in 3D axial view based on the severe case study in Fig. S3.


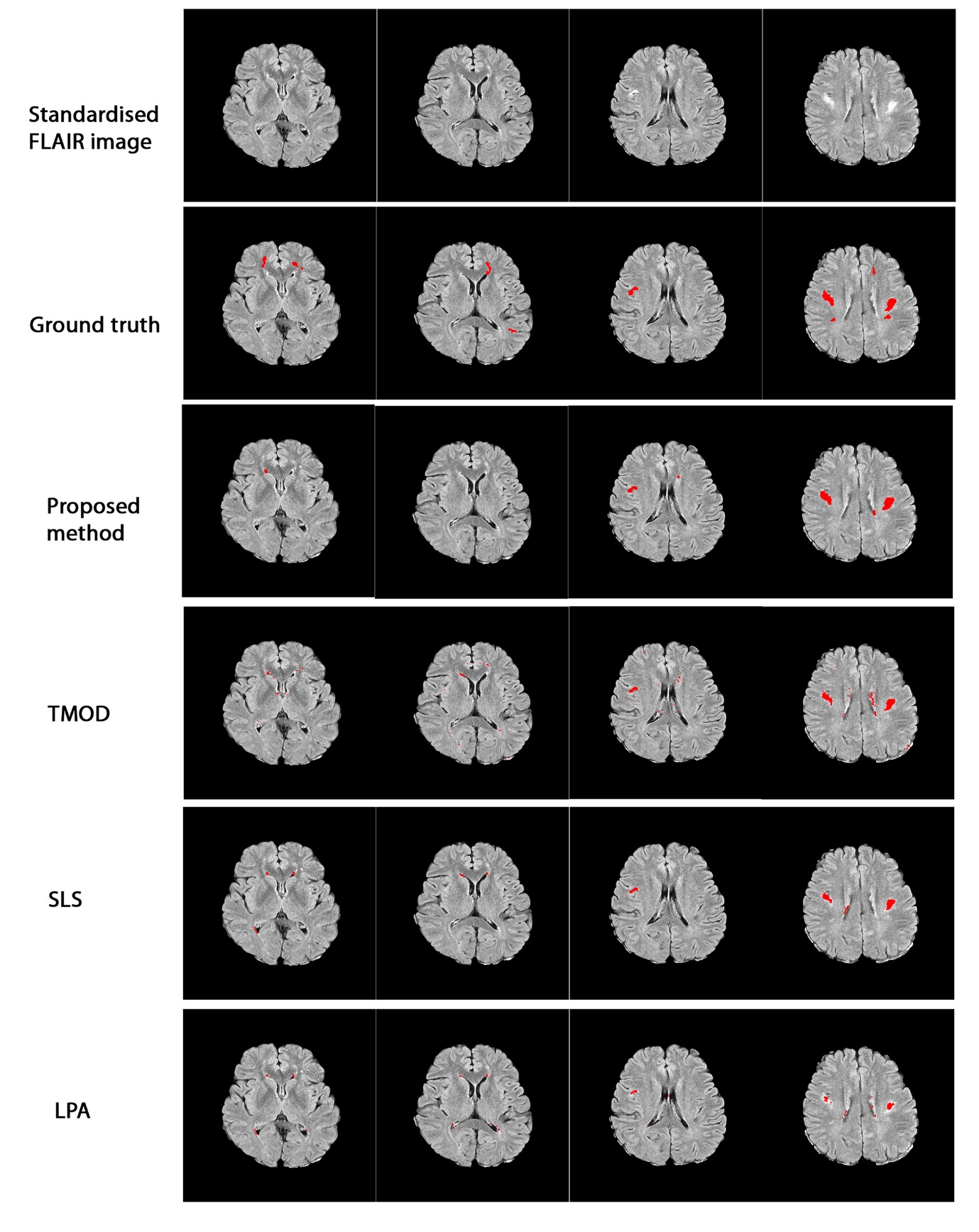


**Fig. S5** Segmented WML is superimposed on top of FLAIR image for the mild case study (< 5mL).


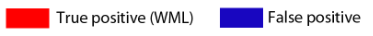


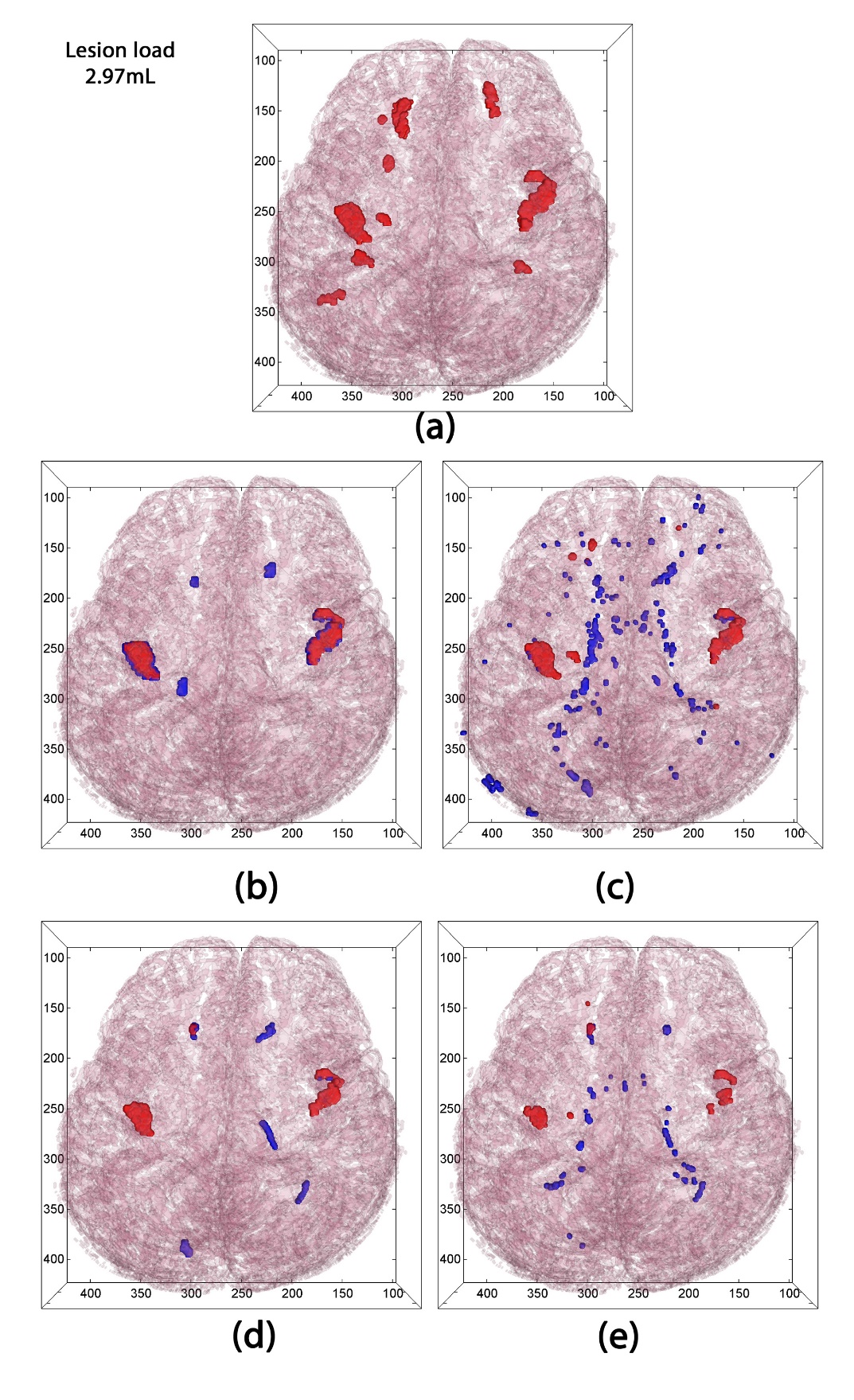


**Fig. S6** Qualitative segmentation results by (a) the neuroradiologist and performance of (b) proposed method, (c) TMOD, (d) SLS, (e) LPA can be visualised in 3D axial view based on the mild case study in Fig. S5.

**Section 2: Top rank methods and recent state-of-the-art works**

In order to perform the validation tests for the proposed method, four states of the art methods were used to compare and quantify WML on the same image datasets in this study. The methods included TMOD [1], SLS (http://atc.udg.edu/salem/slsToolbox/software.html) [2] and LPA (http://www.applied-statistics.de/lst.html) [3]. It is worth noting that SLS[4] and LPA are publicly available lesion segmentation toolboxes; both SLS and LPA run as extension under the Statistical Parametric Mapping platform (SPM12, http://www.fil.ion.ucl.ac.uk/spm/software/spm12/). For fair comparison, image input for three automated approaches would be pre-processed with bias field corrections by default.

Trimmed mean outlier detection is a fully automated WML segmentation[1] performed on T1-w and FLAIR images to segment WML. The T1-w image is used in the skull stripping process to generate the brain mask. Voxels of healthy brain tissues from the FLAIR image are then extracted based on this brain mask. Subsequently, the voxels of healthy brain tissue will be used in a boxplot and trimmed mean analysis to estimate the optimal threshold value for WML (voxel with hyper-intensity). The true lesion is obtained and determined by excluding the FP of the lesion from the region of white matter. The method has been evaluated with an MS dataset from MICCAI challenge 2008. Based on the intensive evaluation, the approach ranked third among other methods with the overall score of 81.9541 at the time of submission.

The algorithm named SALEM-LS is an algorithm first introduced by Roura et al.[2] and the segmentation tool has been validated with the dataset from same challenge. The approach received an overall score of 82.344 and ranked the first among other methods at the time of submission. The publicly available software package published by Roura et al. [4] was used to detect and segment WML with the input of T1-w and FLAIR images in this study. The algorithm adaptively threshold the outliers from grey matter tissues of the FLAIR image. The outliers are defined as $Tr= \mu+ \alpha\sigma,$ where µ and σ are the mean and standard deviation of grey matter intensity distribution respectively. In their work, the $\alpha$ parameter showed significant influence on the performance of the method. In their post-processing rule, λ_ts_ and λ_nb_ were the threshold parameters defined as the WML and neighbourhood of the WML, respectively. We fixed the $\alpha$ parameter with 3, λ_ts_=0.6 and λ_nb_=0.7 for the first iteration. Also, the $\alpha$ parameter with 2, λ_ts_=0.6 and λ_nb_=0.5 were fixed for second iteration as recommended by authors [4] for 1.5T MR datasets acquired by the Signa HDx GE Scanner.

The lesion prediction algorithm is the lesion segmentation algorithm newly developed by Schmidt et al. [3]. The input of LPA is a FLAIR image only (T1-w image is optional), and no initial parameter is required to set when performing the segmentation. The method consists of a binary classifier in the form of a logistic regression model. The model was trained with the pattern of severe WML loads from 53 MS patients. Lesion belief map, a probability map of likelihood that each voxel is part of a lesion, and voxel-specific changes in lesion probability were incorporated in the model. The output of LPA is the lesion probability for each voxel in the image. We fixed the above 0.3 probability as the true lesion based on author suggestion.

**Section 3: Pearson Correction Analysis**

Correlation analysis was employed to measure the correlation between the WML load (volume, mL) detected in the proposed automated WML segmentation and manual delineation by an experienced neuroradiologist across the full range of total loads. A correlation value of 1 indicates the best correlation. The correlation analysis was also applied to all three other algorithms. A significant correlation coefficient (*r* =0.996, *p*-value <0.001) was observed between the proposed method and manual delineation by a neuroradiologist as illustrated in Fig. S7. Although all algorithms showed high and relatively similar *r* values, demonstrating overall close agreement with WML across all sizes, that of the proposed method was numerically the highest (vs. TMOD: *r* =0.994, LPA: *r* =0.966, SLS: *r* =0.993). A logarithmic scale scatter plot is provided as shown in Fig. S8 to better demonstrate the data point distinction.


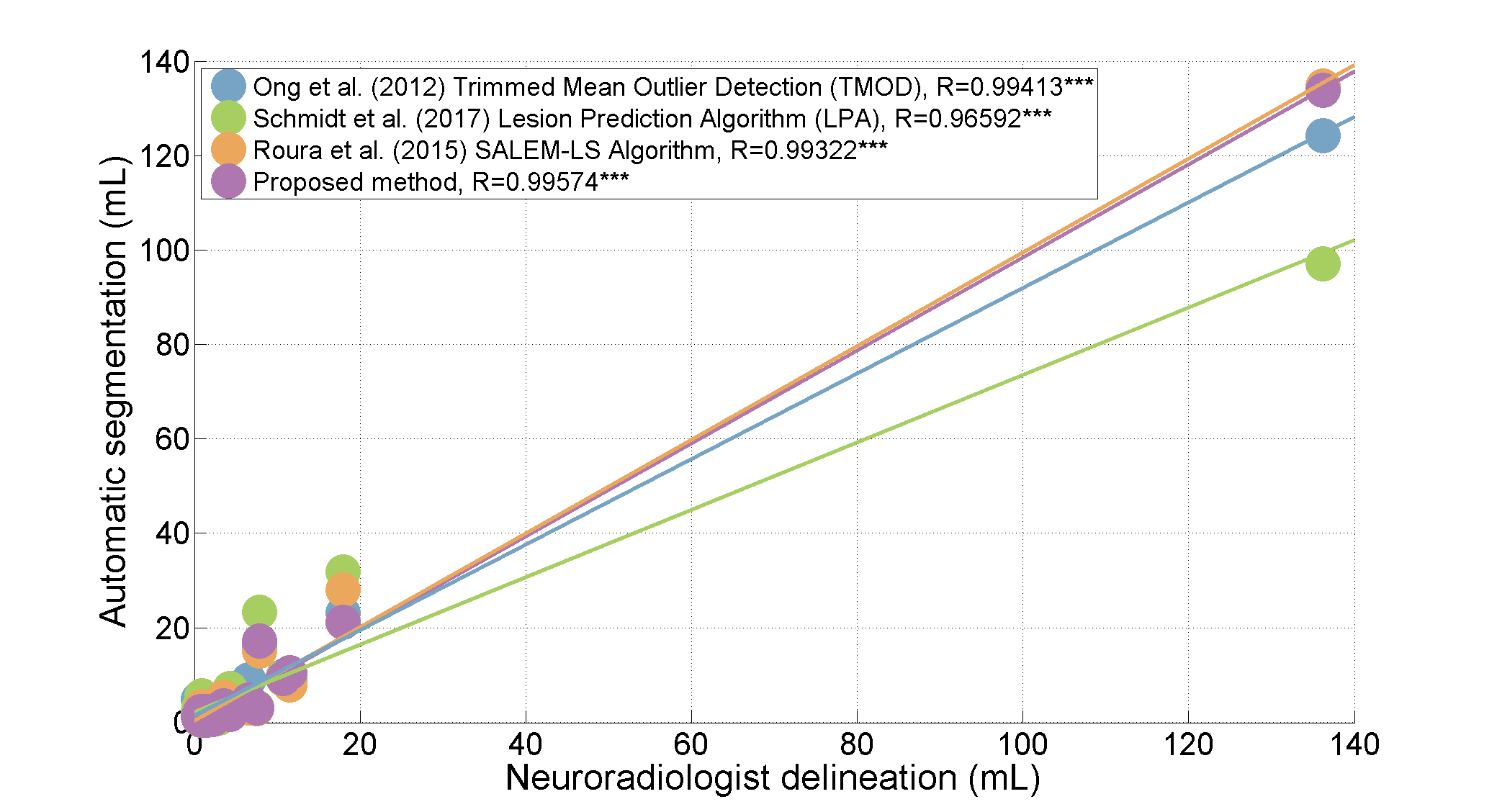


**Fig. S7** A scatter plot of the simple linear regression analysis (32 subjects) for WML volume of each automated method compared to the ground truth.


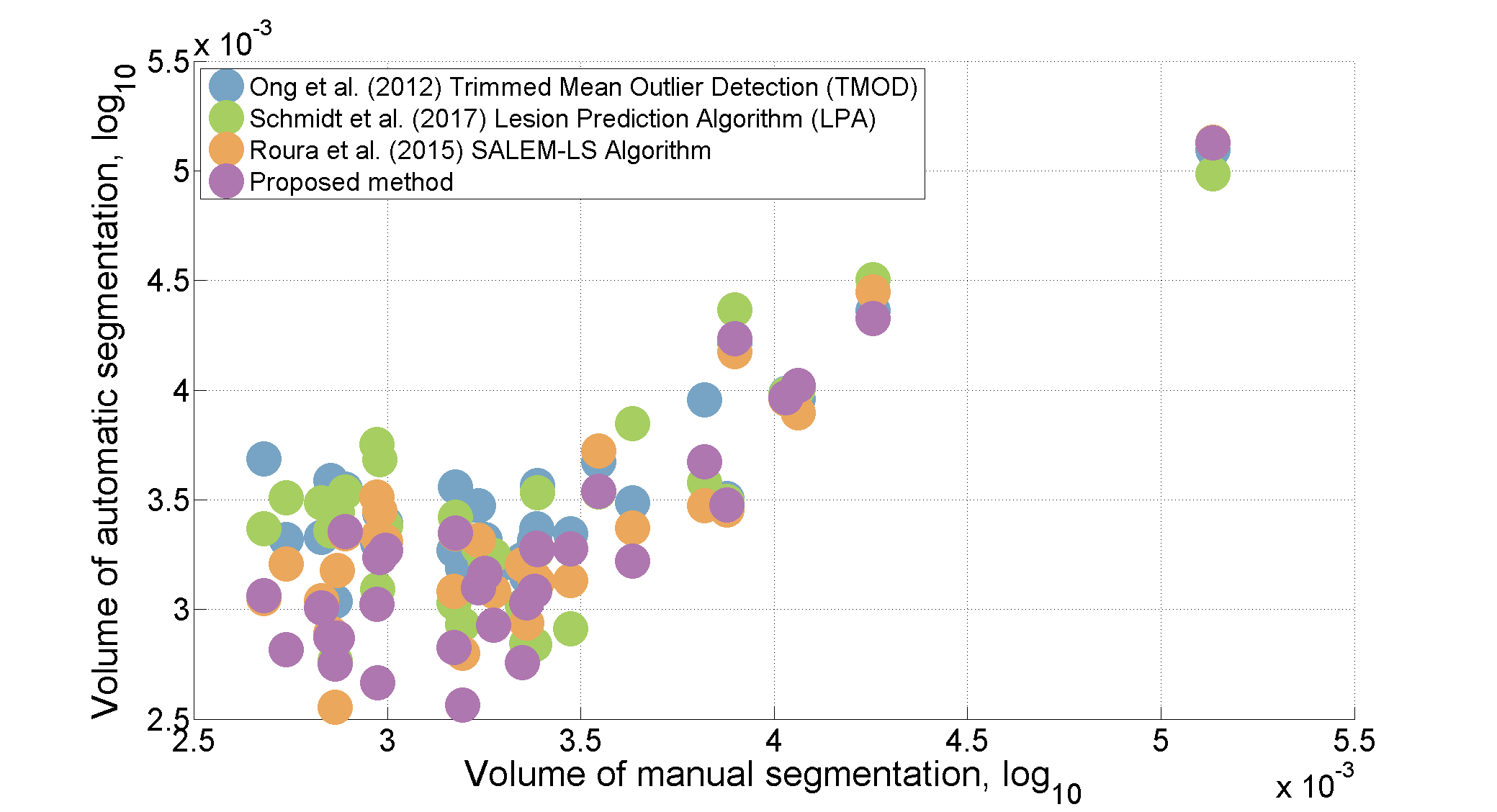


**Fig. S8** A log scale scatter plot (32 subjects) based on WML volume of each automated method compared to the ground truth.

## **Section 4: The optimum parameters of Random Forest Classifier used in the study**

There are two crucial parameters required when performing the WML detection with RF classifier. The parameters are the number of trees, *T*, and tree depth, *D*. The classification model will be more generalised when *T* increases, while if *D* increases, it will lead to over-fitting. Thus, we evaluated the RF classified with the proposed enhanced intensity features and cluster-based texture features by a 10-fold cross-validation on our training dataset to obtain the optimum *T* and *D* value in the model. The detection results were validated for each combination of *T* and *D* parameters with the percentage of instances that were correctly classified, leading to optimal random forest parameters of *T* = 25, and *D* = 25 to avoid under- and over-fitting.


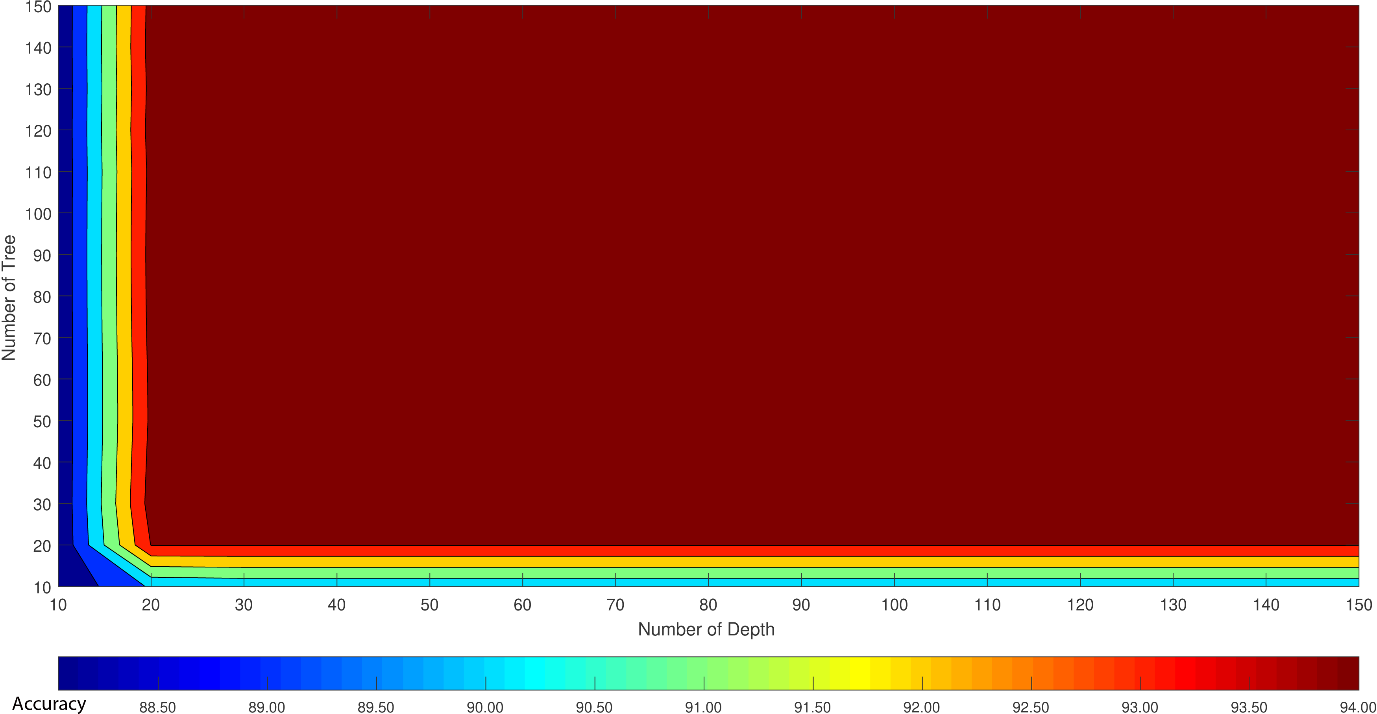


**Fig. S9** Accuracy heat map generated based on number of tree and number of depth under 10-fold cross-validation.

## **Section 5: *k*-means algorithm**

*k*-means algorithm in our implementation is described as follows:

1. Initialise a number of cluster *k* and cluster centre *C_k_* (arbitrary selection).
2. Compute the Euclidean distance *d*, between each cluster centre and each voxel intensity using Eq. 1.

$$d=\left\| I\left( x,y \right)-C_{k} \right\| (1)$$

1. Assign each voxel intensity to the nearest centre based on distance *d*.
2. Once all voxels intensities are completely assigned to cluster *k*, recompute and update coordinate location of centre based on Eq. 2.

$$C_{k}=\frac{1}{k}\sum\sum I\left( x,y \right)\text{ and }I\in C_{k} (2)$$

3. Repeat the process until it satisfies the sum of squared within cluster errors defined as Eq. 3.

$$J=\sum\sum\left\| I\left( x,y \right)-C_{k} \right\|^{2}\text{ }\text{and }I\in C_{k} (3)$$

**Table S1** Agreement measures for Dice index, Jaccard index, positive predictive value, true positive rate, false positive rate, and volume difference between lesions delineated by the automated methods and two neuroradiologist for 25 subjects with mild WML loads.

| Automated Segmentation methods | Annotator | Dice Index  (B=1, W=0) | Jaccard Index  (B=1, W=0) | Positive Predictive Value  (B=1, W=0) | True Positive Rate  (B=1, W=0) | False Positive Rate  (B=0, W=1) | Volume Difference  (B=0, W=∞) |
| --- | --- | --- | --- | --- | --- | --- | --- |
| Proposed method | Neurorad 1  Neurorad 2 | 0.2648 ±0.134  0.2585±0.129 | 0.1598±0.098  0.1550±0.093 | 0.3383±0.218  0.3390±0.218 | 0.2555±0.143  0.2463±0.137 | 0.3656±0.1890  0.3584±0.192 | 0.5056±0.435  0.5256±0.424 |
| TMOD | Neurorad 1  Neurorad 2 | 0.2397± 0.123  NS  0.2367±0.120  NS | 0.1420±0.087  NS  0.1398±0.085  NS | 0.2180±0.139  **  0.2204±0.143  ** | 0.3351±0.150  *  0.3249±0.144  * | 0.5594±0.1910  ***  0.5498±0.197  *** | 1.4350±2.005  **  1.4090±2.008  ** |
| SLS | Neurorad 1  Neurorad 2 | 0.1471± 0.131  ***  0.1444±0.126  *** | 0.0851±0.085  **  0.0830±0.080  ** | 0.1680±0.167  ***  0.1696±0.168  *** | 0.1609±0.148  **  0.1539±0.135  ** | 0.4748±0.1959  *  0.4660±0.198  * | 0.7978±0.653  *  0.7854±0.631  * |
| LPA | Neurorad 1  Neurorad 2 | 0.1275± 0.080  ***  0.1251±0.077  *** | 0.0700±0.047  ***  0.0685±0.045  ** | 0.1331±0.122  ***  0.1341±0.123  *** | 0.1913±0.157  *  0.1855±0.155  * | 0.5417±0.2287  **  0.5330±0.233  ** | 1.4973±1.673  **  1.4971±1.641  ** |

The first line represents neuroradiologist 1, and the second line represents neuroradiologist 2; Dice index, Jaccard index, positive predictive value, true positive rate, false positive rate, and volume difference are presented as mean ±standard deviation; B indicates Best, and W is Worst; NS is not statistically significantly different compared with the proposed method (P>0.05); * is significant at P ≤ 0.05; ** is significant at P ≤ 0.01, and *** is significant at P ≤ 0.001; All paired t-tests were Bonferroni corrected for four comparisons.

**Table S2** Agreement measures for Dice index, Jaccard index, positive predictive value, true positive rate, false positive rate, and volume difference between lesions delineated by the automated methods and two neuroradiologist for 5 subjects with moderate WML loads.

| Automated Segmentation methods | Annotator | Dice Index  (B=1, W=0) | Jaccard Index  (B=1, W=0) | Positive Predictive Value  (B=1, W=0) | True Positive Rate (B=1, W=0) | False Positive Rate  (B=0, W=1) | Volume Difference  (B=0, W=∞) |
| --- | --- | --- | --- | --- | --- | --- | --- |
| Proposed method | Neurorad 1  Neurorad 2 | 0.4547±0.150  0.4745±0.134 | 0.3031±0.115  0.3239±0.118 | 0.4914±0.130  0.5253±0.153 | 0.4736±0.230  0.4879±0.215 | 0.2262±0.187  0.2970±0.162 | 0.4569±0.446  0.4646±0.416 |
| TMOD | Neurorad 1  Neurorad 2 | 0.4548±0.138  NS  0.4792±0.142  NS | 0.3021±0.109  NS  0.3189±0.112  NS | 0.4701±0.132  NS  0.4947±0.165  NS | 0.4945±0.223  NS  0.5049±0.207  NS | 0.2181±0.215  NS  0.3357±0.165  NS | 0.4645±0.387  NS  0.4417±0.376  NS |
| SLS | Neurorad 1  Neurorad 2 | 0.4065±0.196  NS  0.4154±0.196  NS | 0.2694±0.146  NS  0.2767±0.148  NS | 0.4724±0.189  NS  0.4923±0.208  NS | 0.4039±0.263  NS  0.4071±0.259  NS | 0.2642±0.268  NS  0.2724±0.156  NS | 0.5088±0.290  NS  0.5096±0.273  NS |
| LPA | Neurorad 1  Neurorad 2 | 0.3593±0.169  NS  0.3751±0.174  NS | 0.2288±0.119  NS  0.2418±0.127  NS | 0.3808±0.159  NS  0.4053±0.184  NS | 0.4095±0.281  NS  0.4182±0.277  NS | 0.2567±0.349  NS  0.3476±0.188  NS | 0.6371±0.758  NS  0.6412±0.742  NS |

The first line represents neuroradiologist 1, and the second line represents neuroradiologist 2; Dice index, Jaccard index, positive predictive value, true positive rate, false positive rate, and volume difference are presented as mean ±standard deviation; B indicates Best, and W is Worst; NS is not statistically significantly different compared with the proposed method (P>0.05); * is significant at P ≤ 0.05; ** is significant at P ≤ 0.01, and *** is significant at P ≤ 0.001; All paired t-tests were Bonferroni corrected for four comparisons.

**Table S3** Agreement measures for Dice index, Jaccard index, positive predictive value, true positive rate, false positive rate, and volume difference between lesions delineated by the automated methods and two neuroradiologist for 2 subjects with Severe WML loads.

| Automated Segmentation methods | Annotator | Dice Index  (B=1, W=0) | Jaccard Index  (B=1, W=0) | Positive Predictive Value  (B=1, W=0) | True Positive Rate  (B=1, W=0) | False Positive Rate  (B=0, W=1) | Volume Difference  (B=0, W=∞) |
| --- | --- | --- | --- | --- | --- | --- | --- |
| Proposed method | Neurorad 1  Neurorad 2 | 0.7263±0.228  0.7239±0.231 | 0.5958±0.286  0.5935±0.289 | 0.7092±0.264  0.7092±0.264 | 0.7472±0.187  0.7416±0.195 | 0.2262±0.189  0.2160±0.212 | 0.0975±0.112  0.0867±0.097 |
| TMOD | Neurorad 1  Neurorad 2 | 0.7455±0.190  NS  0.7430±0.194  NS | 0.6128±0.245  NS  0.6103±0.248  NS | 0.7327±0.268  NS  0.7327±0.268  NS | 0.7699±0.101  NS  0.7635±0.110  NS | 0.2181±0.215  NS  0.2241±0.186  NS | 0.1870±0.140  NS  0.1752±0.124  NS |
| SLS | Neurorad 1  Neurorad 2 | 0.7415±0.243  NS  0.7394±0.245  NS | 0.6192±0.312  NS  0.6172±0.315  NS | 0.6929±0.318  NS  0.6929±0.318  NS | 0.8186±0.126  NS  0.8119±0.136  NS | 0.2642±0.268  NS  0.2619±0.264  NS | 0.2851±0.388  NS  0.2708±0.367  NS |
| LPA | Neurorad 1  Neurorad 2 | 0.6826±0.195  NS  0.6816±0.196  NS | 0.5350±0.227  NS  0.5341±0.229  NS | 0.7063±0.396  NS  0.7069±0.395  NS | 0.7283±0.036  NS  0.7225±0.028  NS | 0.2567±0.349  NS  0.2541±0.345  NS | 0.5280±0.340  NS  0.5118±0.31  NS |

The first line represents neuroradiologist 1, and the second line represents neuroradiologist 2; Dice index, Jaccard index, positive predictive value, true positive rate, false positive rate, and volume difference are presented as mean ±standard deviation; B indicates Best, and W is Worst; NS is not statistically significantly different compared with the proposed method (P>0.05); * is significant at P ≤ 0.05; ** is significant at P ≤ 0.01, and *** is significant at P ≤ 0.001; All paired t-tests were Bonferroni corrected for four comparisons.

**References**

1. Ong KH, Ramachandram D, Mandava R, Shuaib IL: **Automatic white matter lesion segmentation using an adaptive outlier detection method**. *Magnetic Resonance Imaging* 2012, **30**(6):807-823.

2. Roura E, Oliver A, Cabezas M, Valverde S, Pareto D, Vilanova JC, Ramió-Torrentà L, Rovira À, Lladó X: **A toolbox for multiple sclerosis lesion segmentation**. *Neuroradiology* 2015, **57**(10):1031-1043.

3. Schmidt P: **Bayesian inference for structured additive regression models for large-scale problems with applications to medical imaging**. Ludwig-Maximilians-Universität München; 2017.

4. Roura E, Oliver A, Cabezas M, Valverde S, Pareto D, Vilanova JC, Ramió-Torrentà L, Rovira À, Lladó X: **An SPM12 extension for multiple sclerosis lesion segmentation**. In: *SPIE Medical Imaging: 2016*: SPIE; 2016: 6.
